# Supplementary figures and images for: UGGT1-mediated reglucosylation of N-glycan competes with ER-associated degradation of unstable and misfolded glycoproteins
Source: eLife. 2024 Dec 10;12:RP93117. doi: 10.7554/eLife.93117 (PMC11630818; doi:10.7554/eLife.93117)

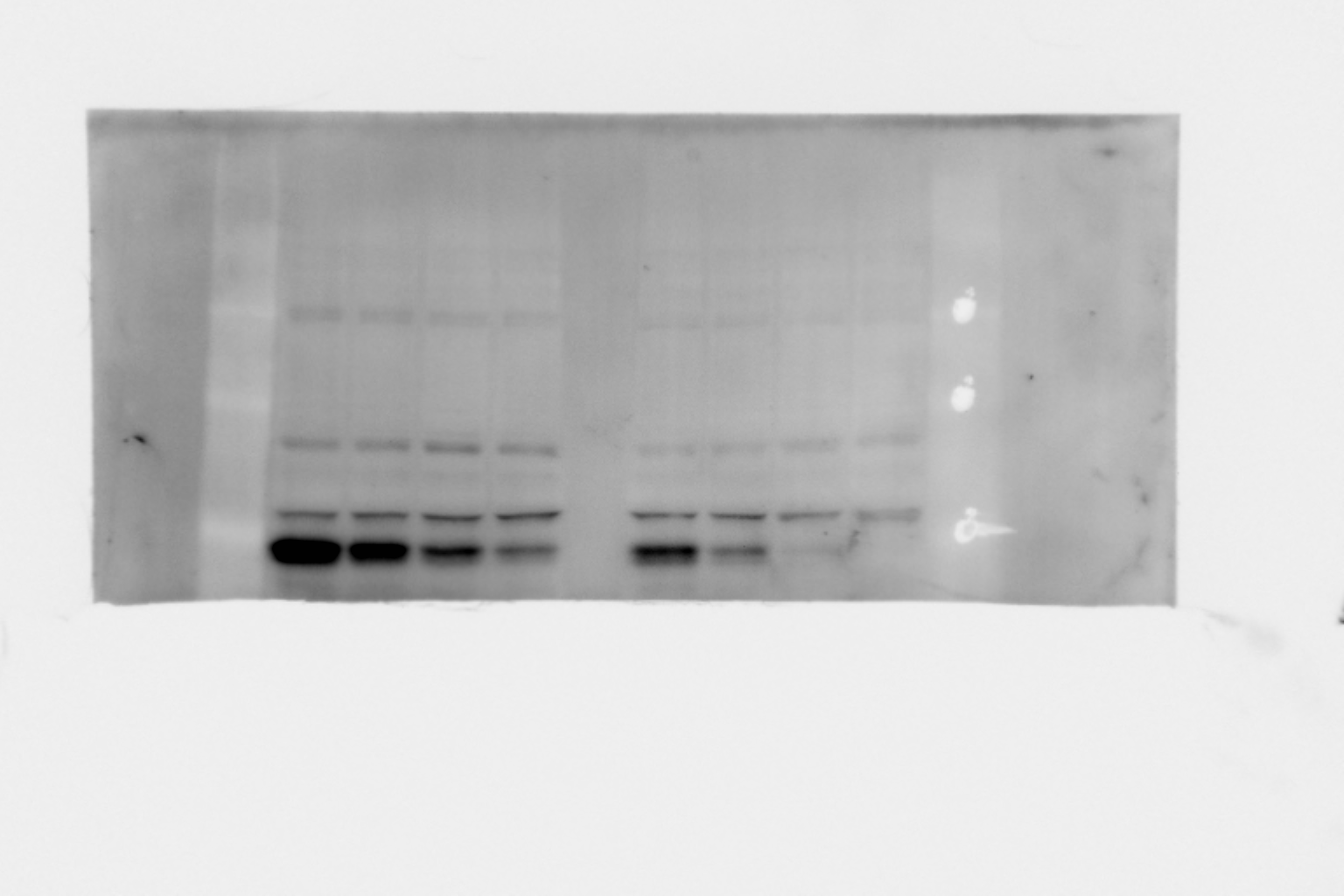

Supplement: Figure 1—source data 1. [file elife-93117-fig1-data1.zip › Fig. 1-Source data 1/Fig.1E-1-2-Source data 1.tif]

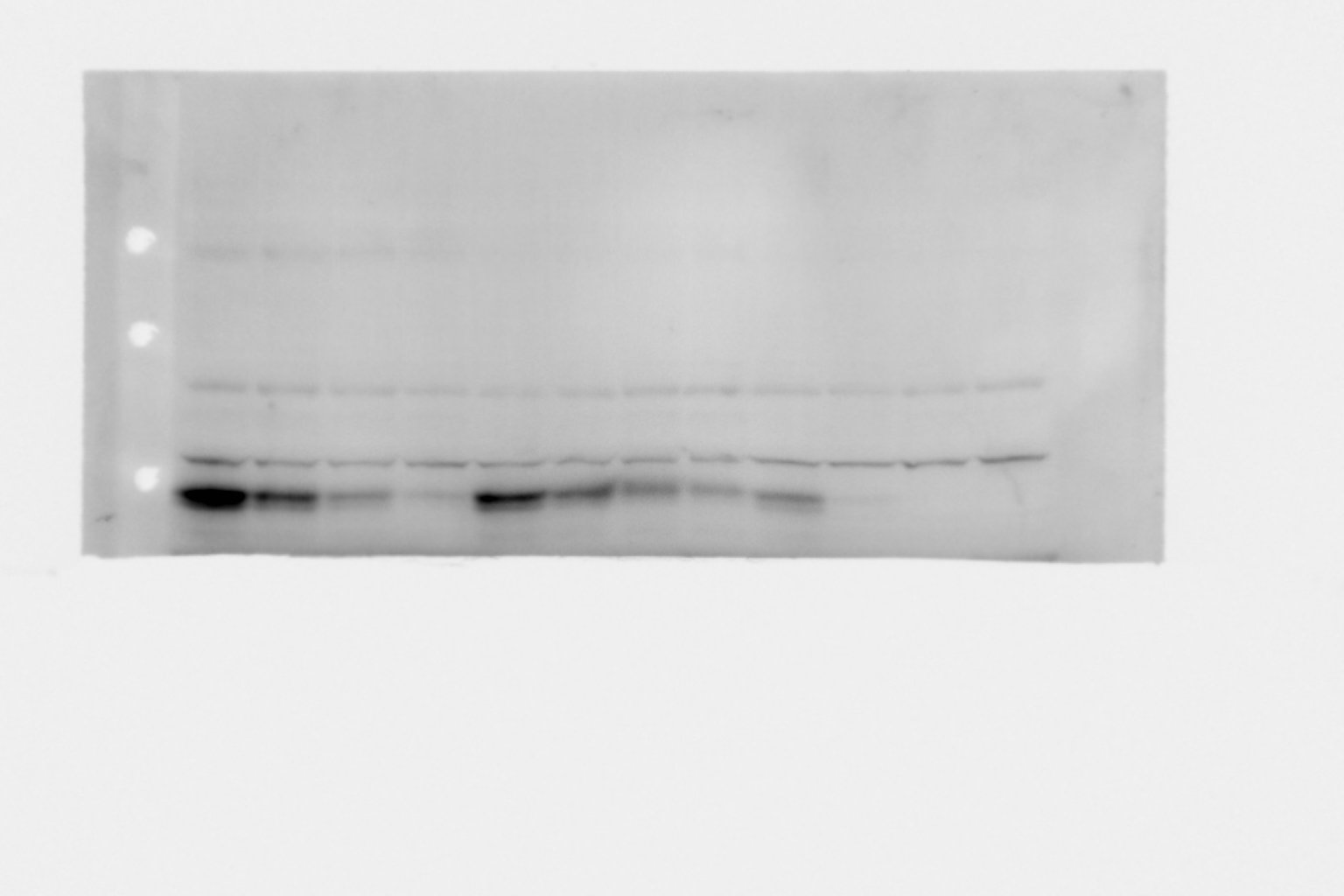

Supplement: Figure 1—source data 1. [file elife-93117-fig1-data1.zip › Fig. 1-Source data 1/Fig.1E-3-4-Source data 1.tif]

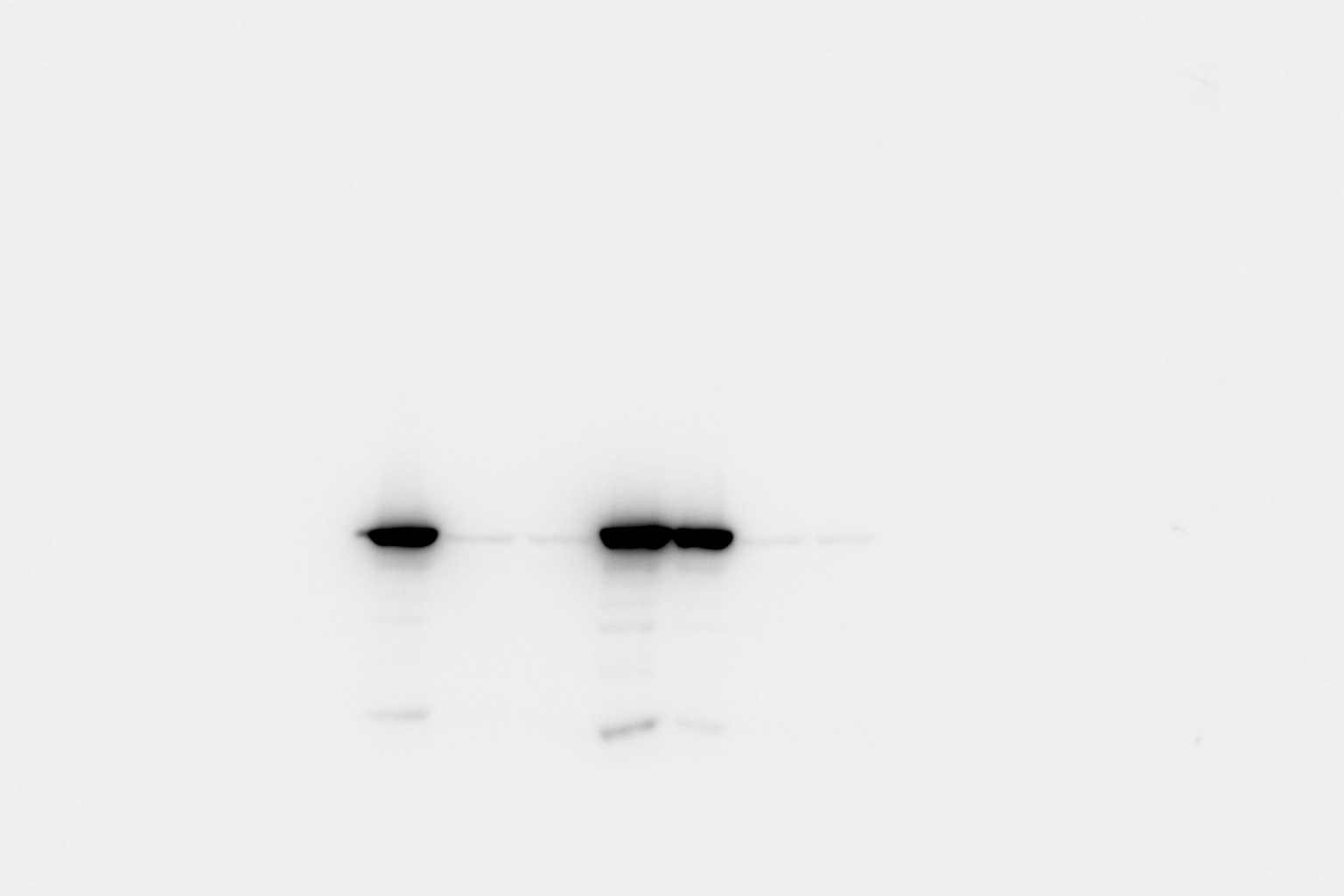

Supplement: Figure 1—source data 1. [file elife-93117-fig1-data1.zip › Fig. 1-Source data 1/Fig.1B-1-Source data 1.tif]

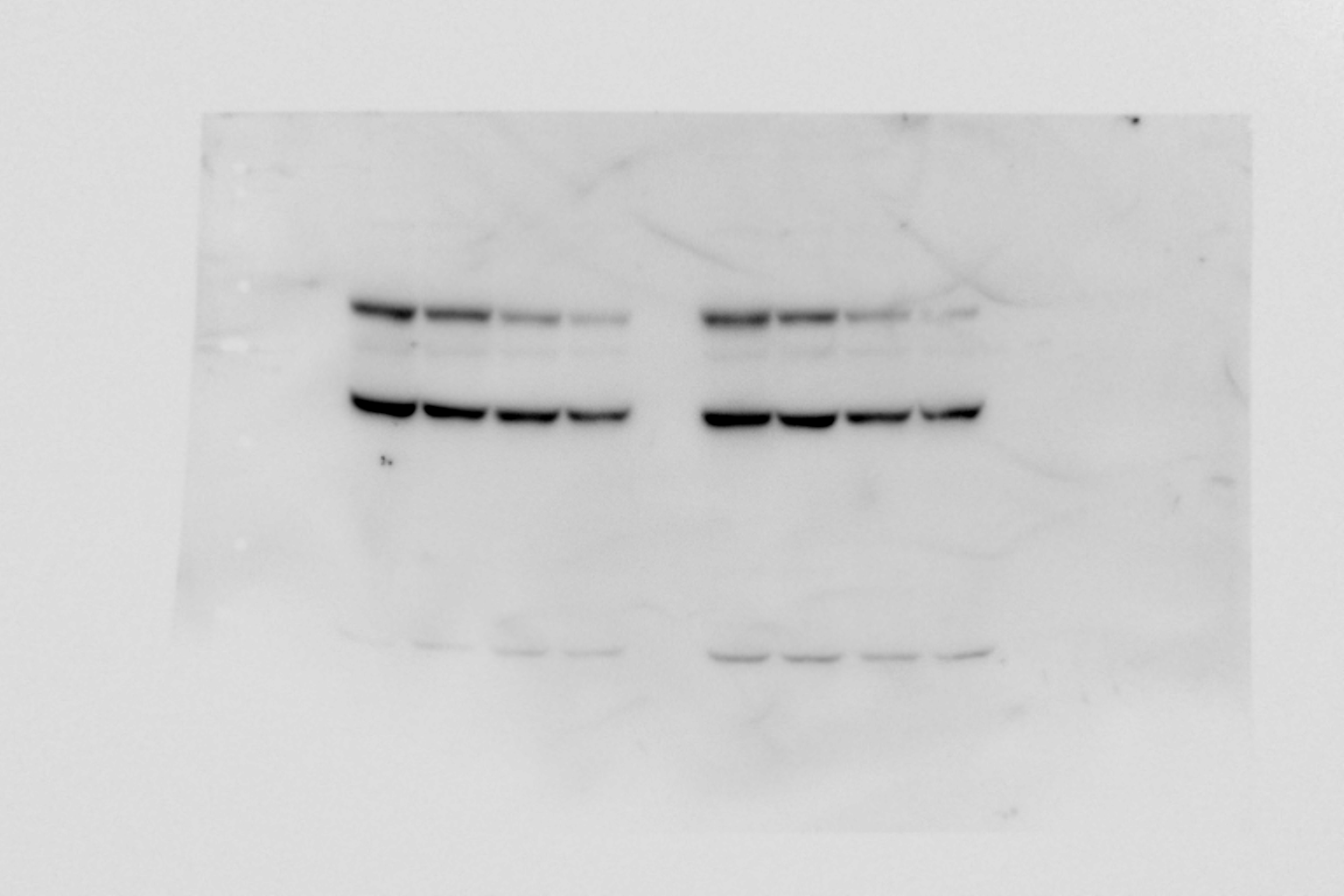

Supplement: Figure 1—source data 1. [file elife-93117-fig1-data1.zip › Fig. 1-Source data 1/Fig.1D-1-2-Source data 1.tif]

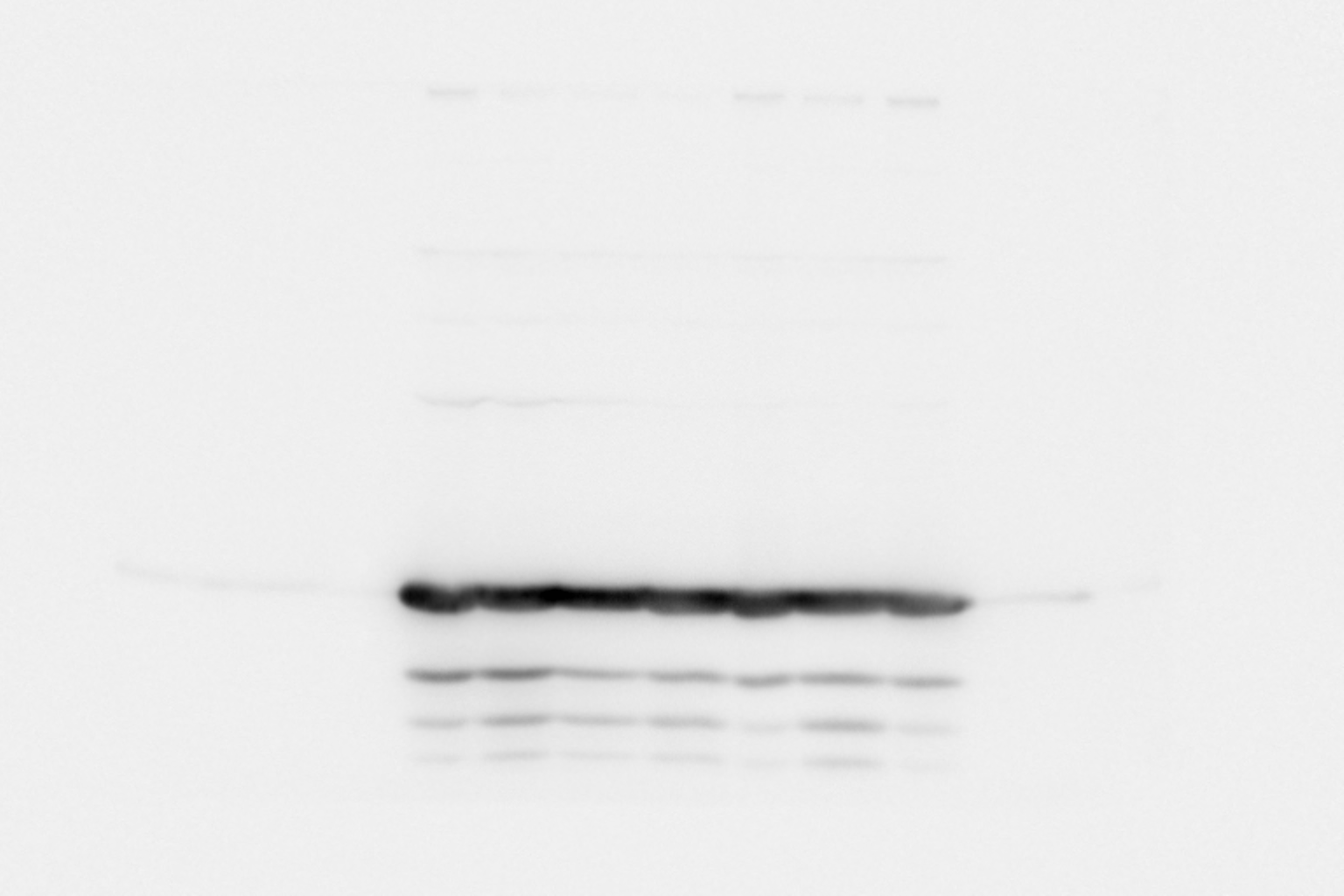

Supplement: Figure 1—source data 1. [file elife-93117-fig1-data1.zip › Fig. 1-Source data 1/Fig.1B-3-Source data 1.tif]

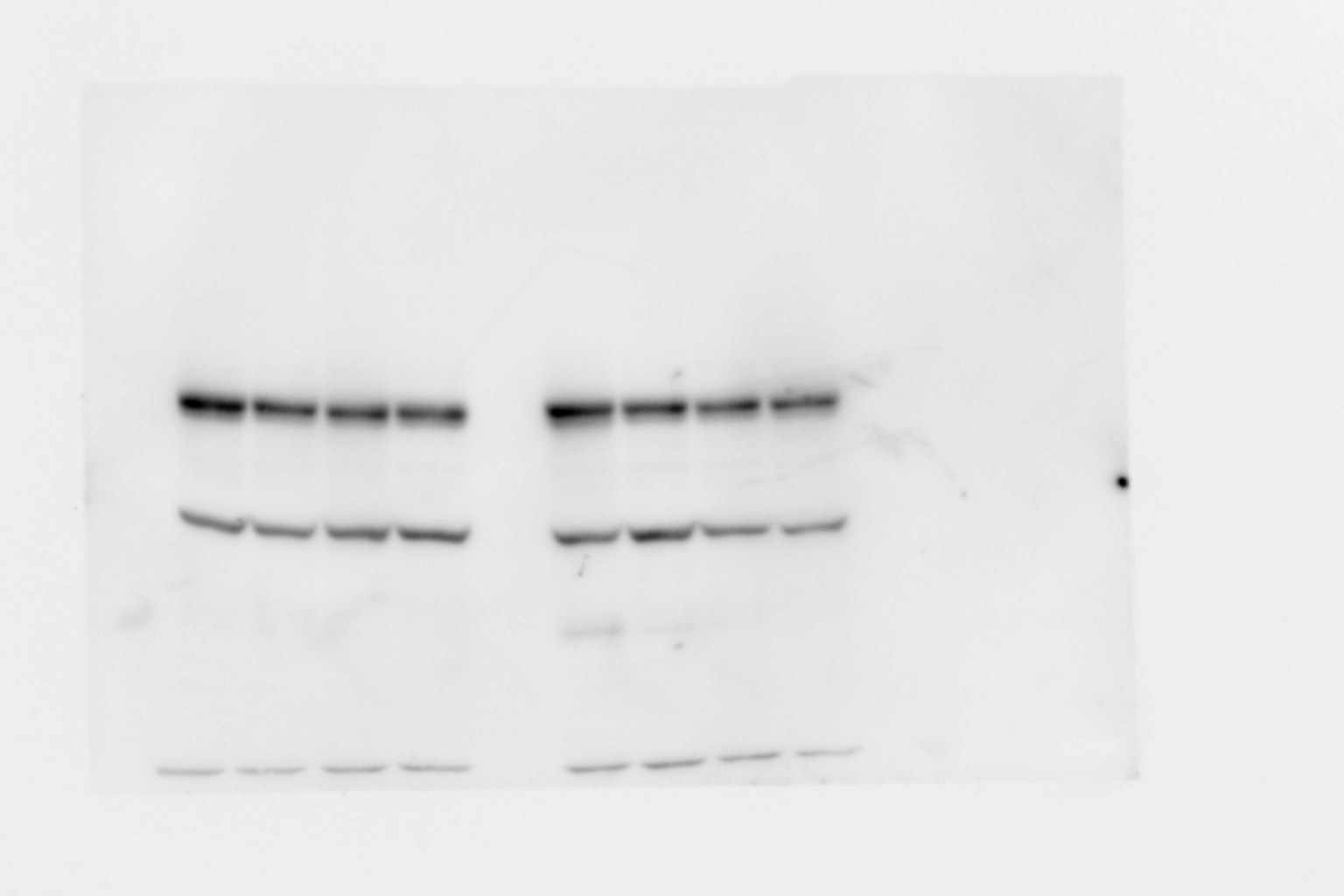

Supplement: Figure 1—source data 1. [file elife-93117-fig1-data1.zip › Fig. 1-Source data 1/Fig.1D-3-4-Source data 1.tif]

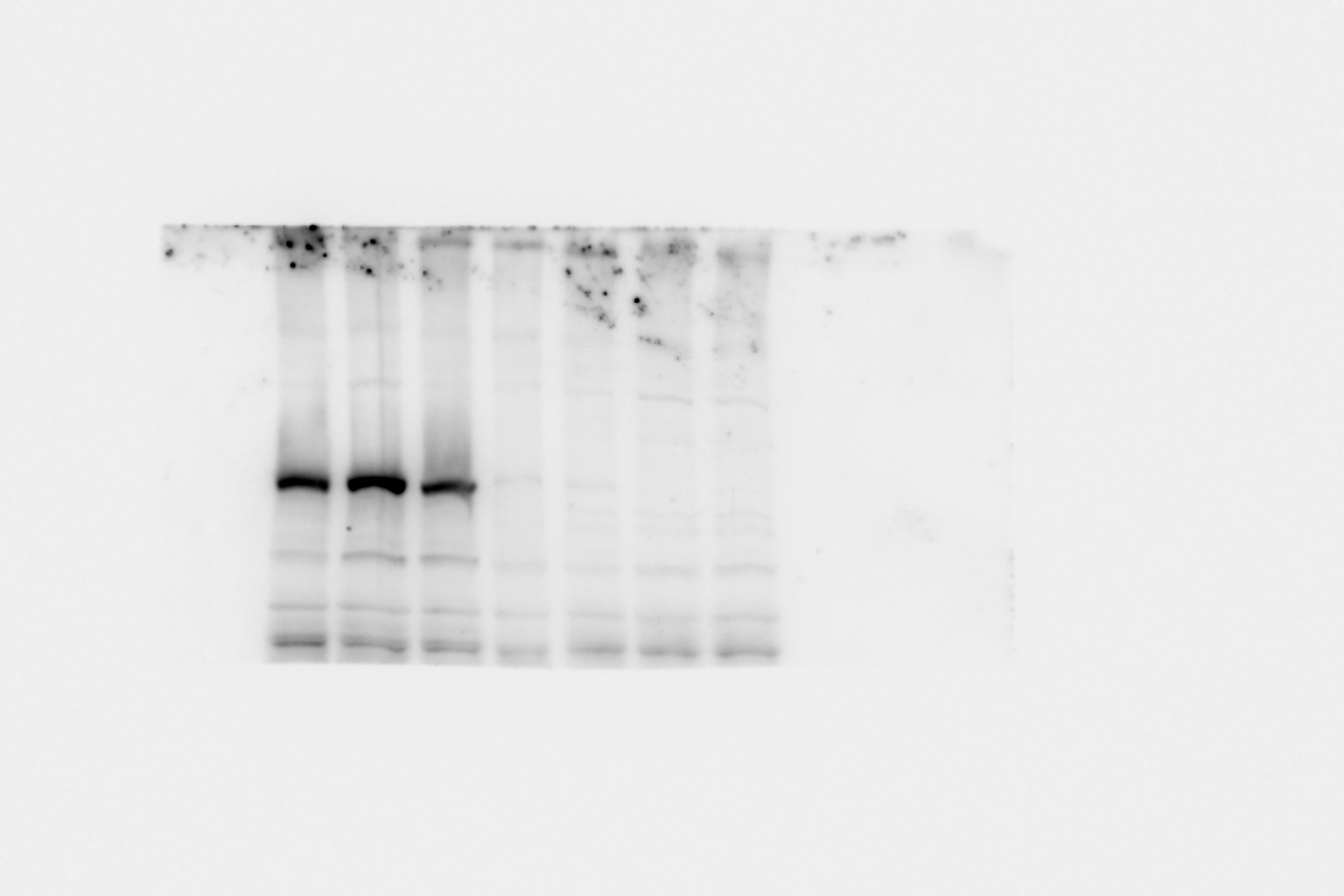

Supplement: Figure 1—source data 1. [file elife-93117-fig1-data1.zip › Fig. 1-Source data 1/Fig.1B-2-Source data 1.tif]

Fig. 1D-H Source data 2 Original membranes corresponding to Fig1D-H  
Fig. 1D

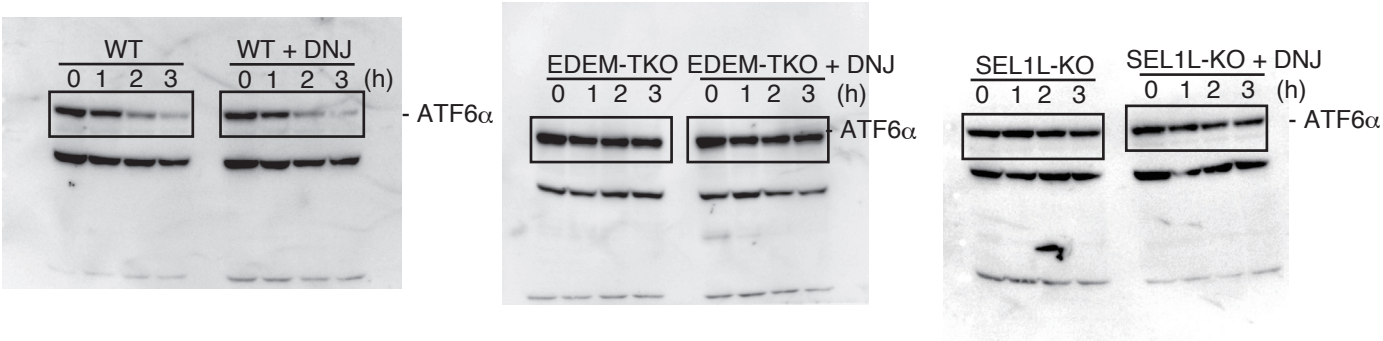

Fig. 1E

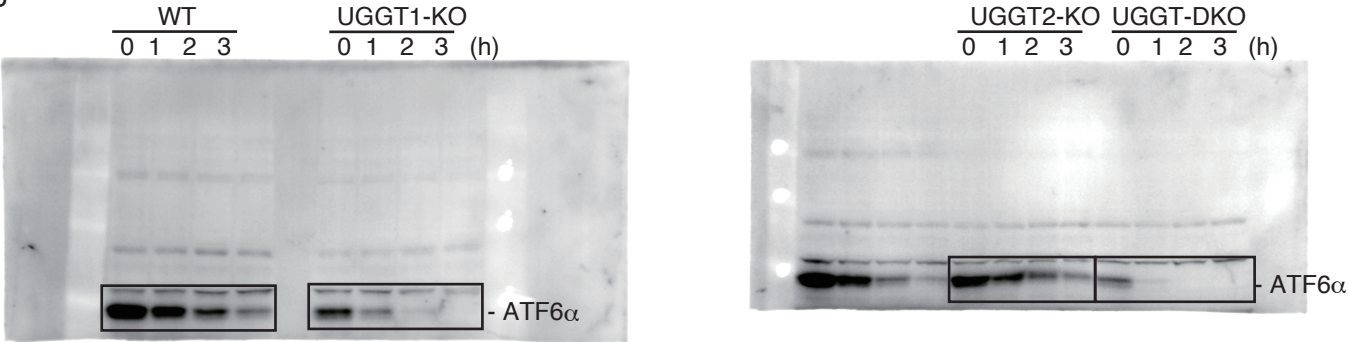

Fig. 1F

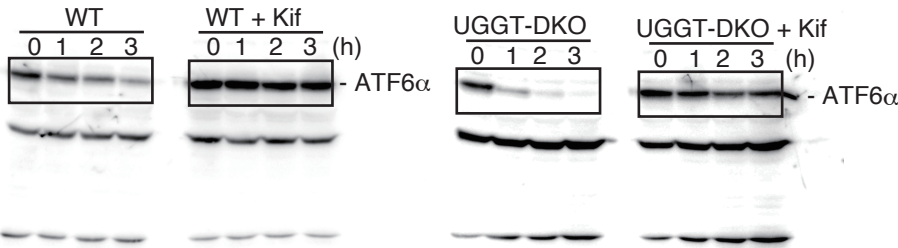

Fig. 1G

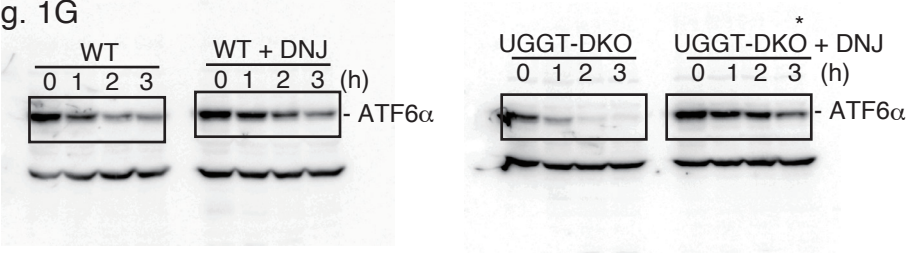

Fig. 1H

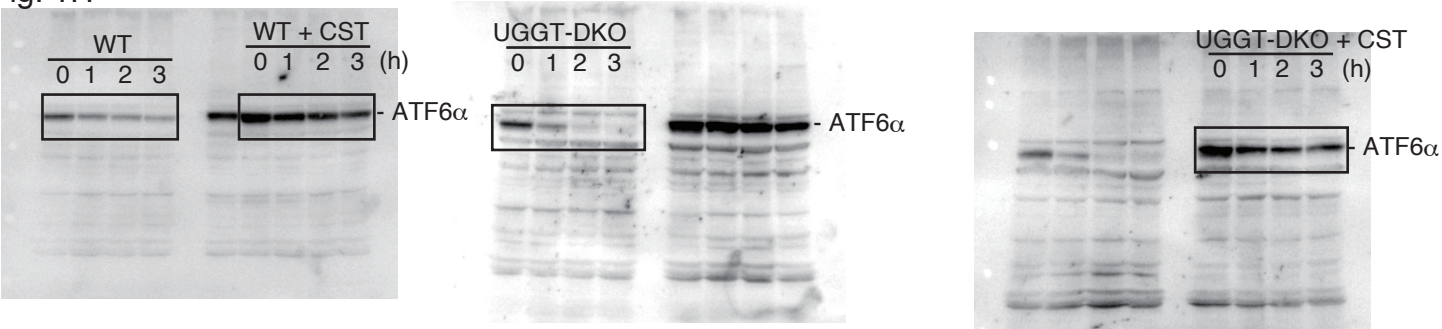

Supplement: Figure 1—source data 2. [file elife-93117-fig1-data2.zip › Fig. 1-Source data 2/Fig. 1D-H-Source data 2.pdf]

Fig. 1B-Source data 1 Original membranes corresponding to Fig1B.

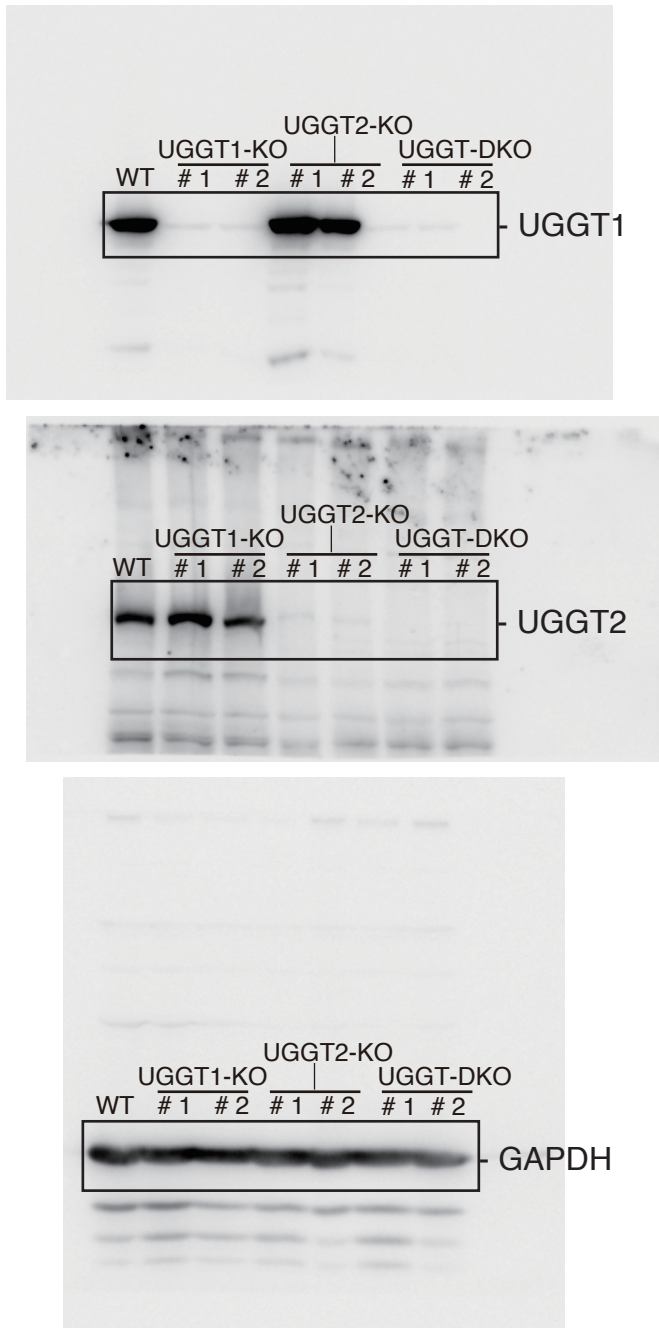

Supplement: Figure 1—source data 2. [file elife-93117-fig1-data2.zip › Fig. 1-Source data 2/Fig. 1B-Source data 2.pdf]

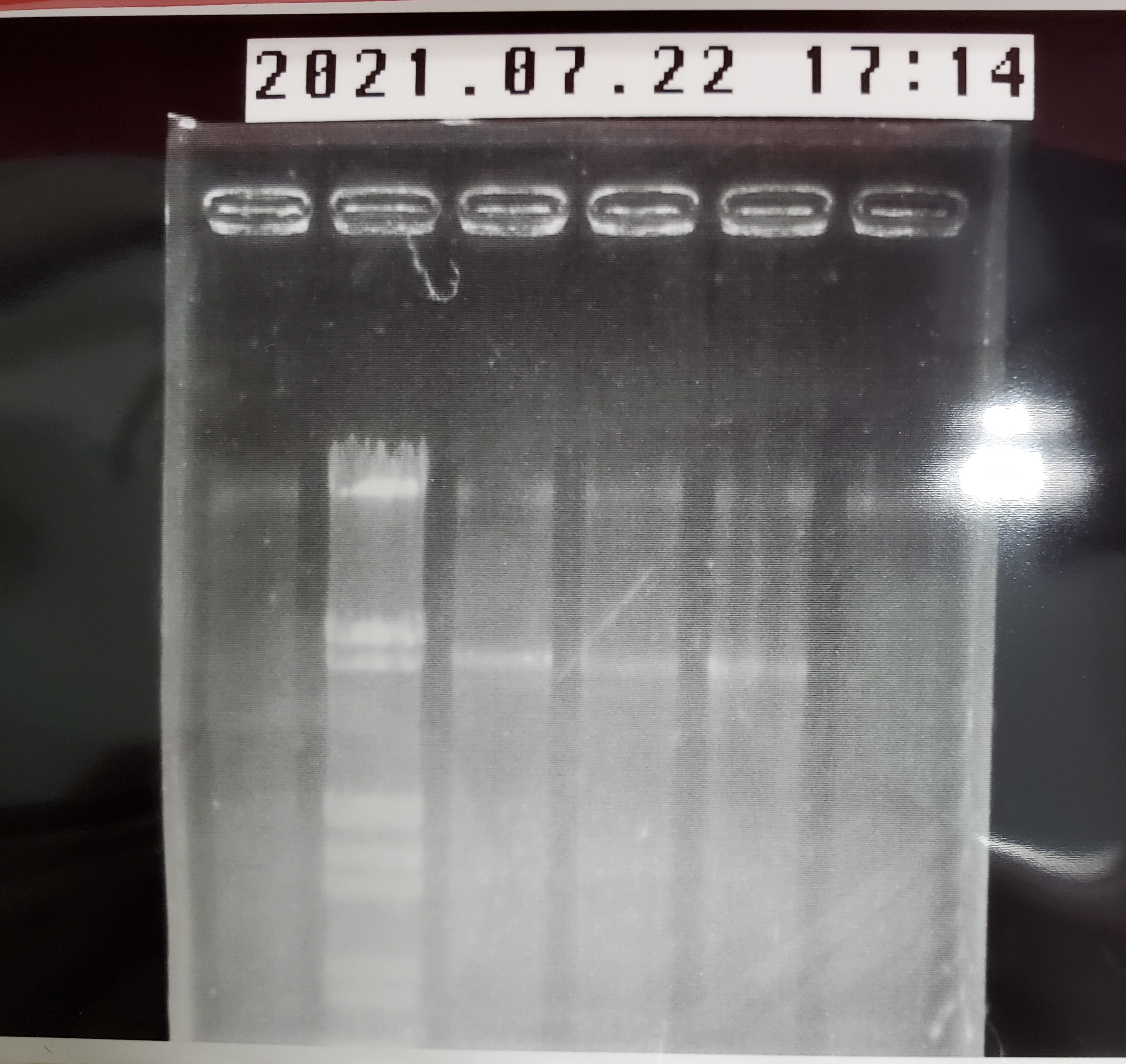

Supplement: Figure 1—figure supplement 1—source data 1. [file elife-93117-fig1-figsupp1-data1.zip › Fig. 1-Figure Supplement-1-Source data 1/Fig.1-Figure Supplement-1H-Source data 1.jpg]

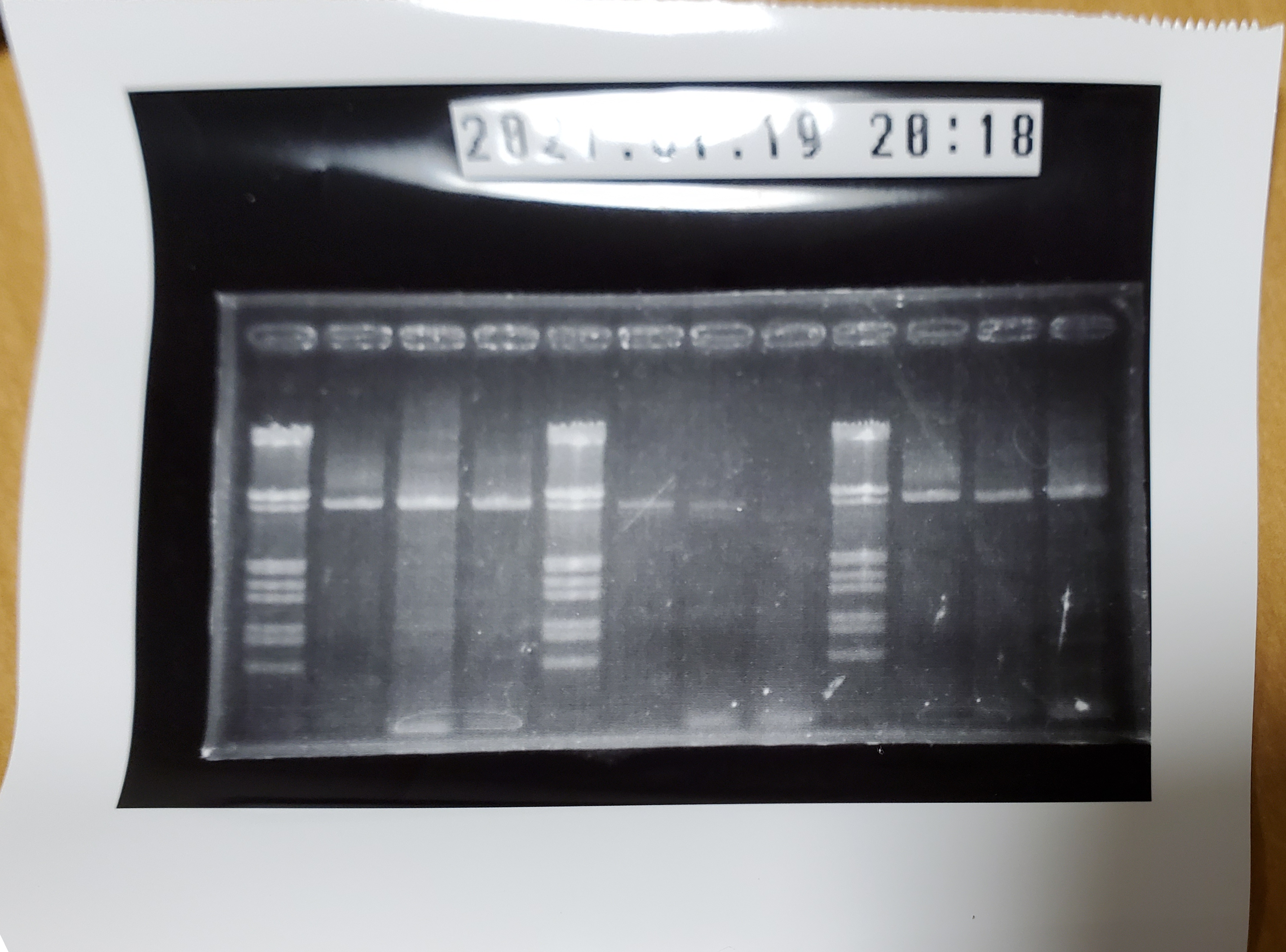

Supplement: Figure 1—figure supplement 1—source data 1. [file elife-93117-fig1-figsupp1-data1.zip › Fig. 1-Figure Supplement-1-Source data 1/Fig.1-Figure Supplement-1G-Source data 1.png]

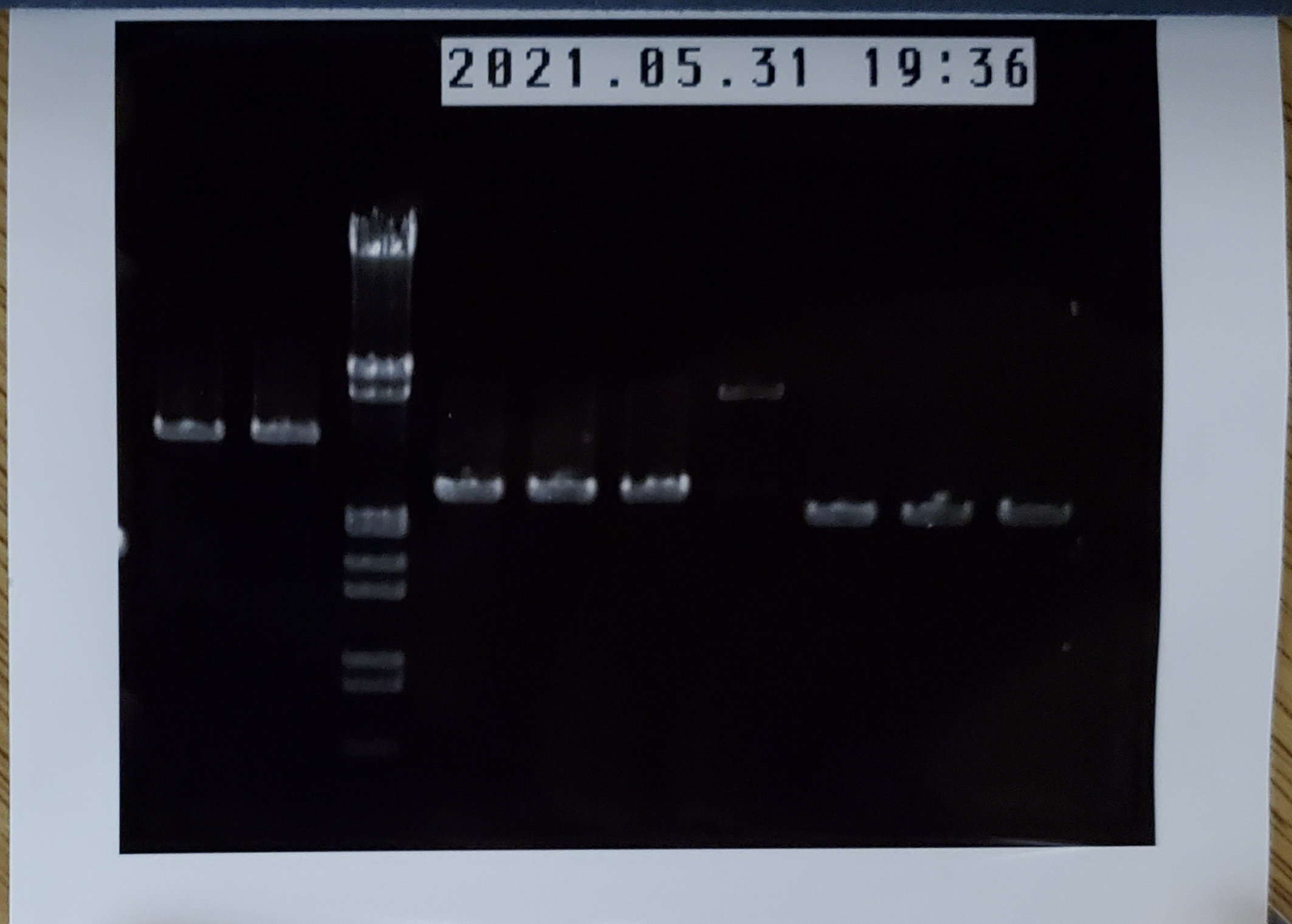

Supplement: Figure 1—figure supplement 1—source data 1. [file elife-93117-fig1-figsupp1-data1.zip › Fig. 1-Figure Supplement-1-Source data 1/Fig.1-Figure Supplement-1F-1-Source data 1.png]

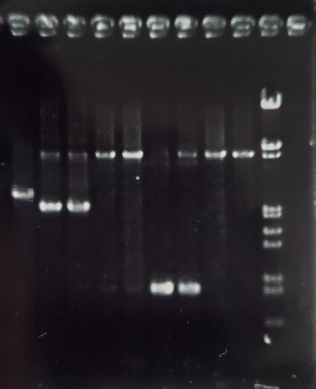

Supplement: Figure 1—figure supplement 1—source data 1. [file elife-93117-fig1-figsupp1-data1.zip › Fig. 1-Figure Supplement-1-Source data 1/Fig.1-Figure Supplement-1F-2-Source data 1.tif]

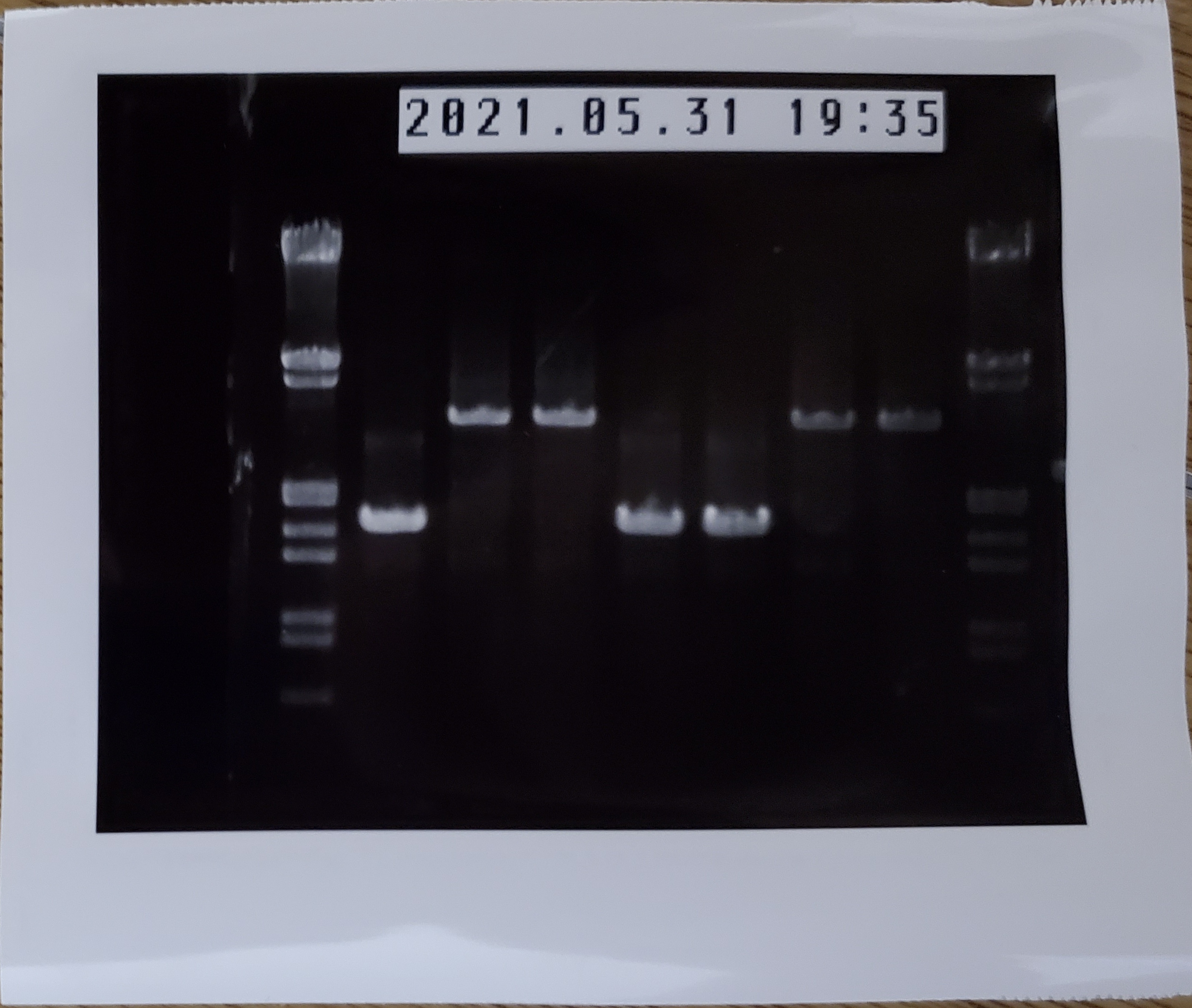

Supplement: Figure 1—figure supplement 1—source data 1. [file elife-93117-fig1-figsupp1-data1.zip › Fig. 1-Figure Supplement-1-Source data 1/Fig.1-Figure Supplement-1E-Source data 1.png]

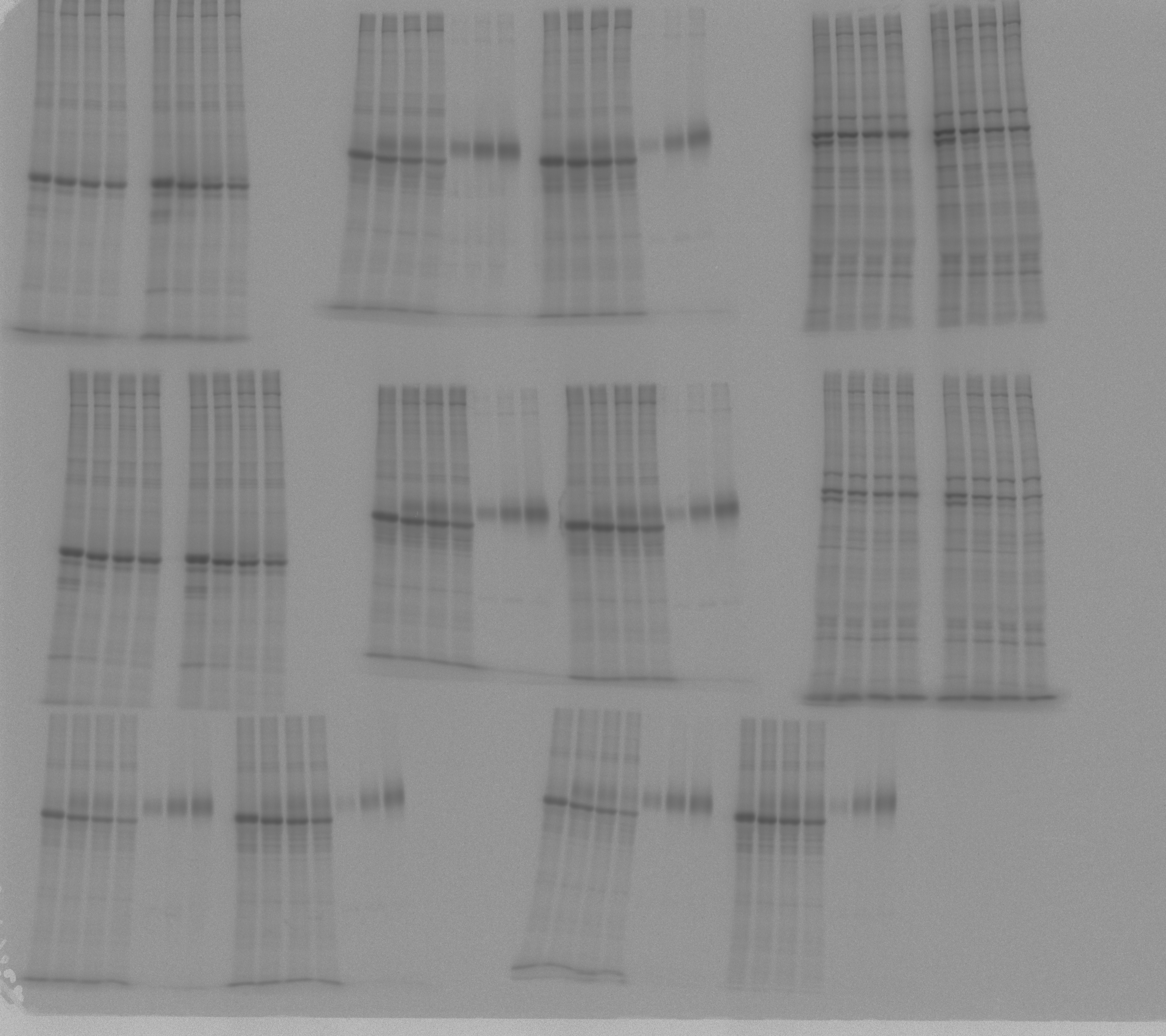

Supplement: Figure 1—figure supplement 2—source data 1. [file elife-93117-fig1-figsupp2-data1.zip › Fig. 1-Figure Supplement-2-Source data 1/Fig.1-Figure Supplement-2D-1-4-Source data 1.TIFF]

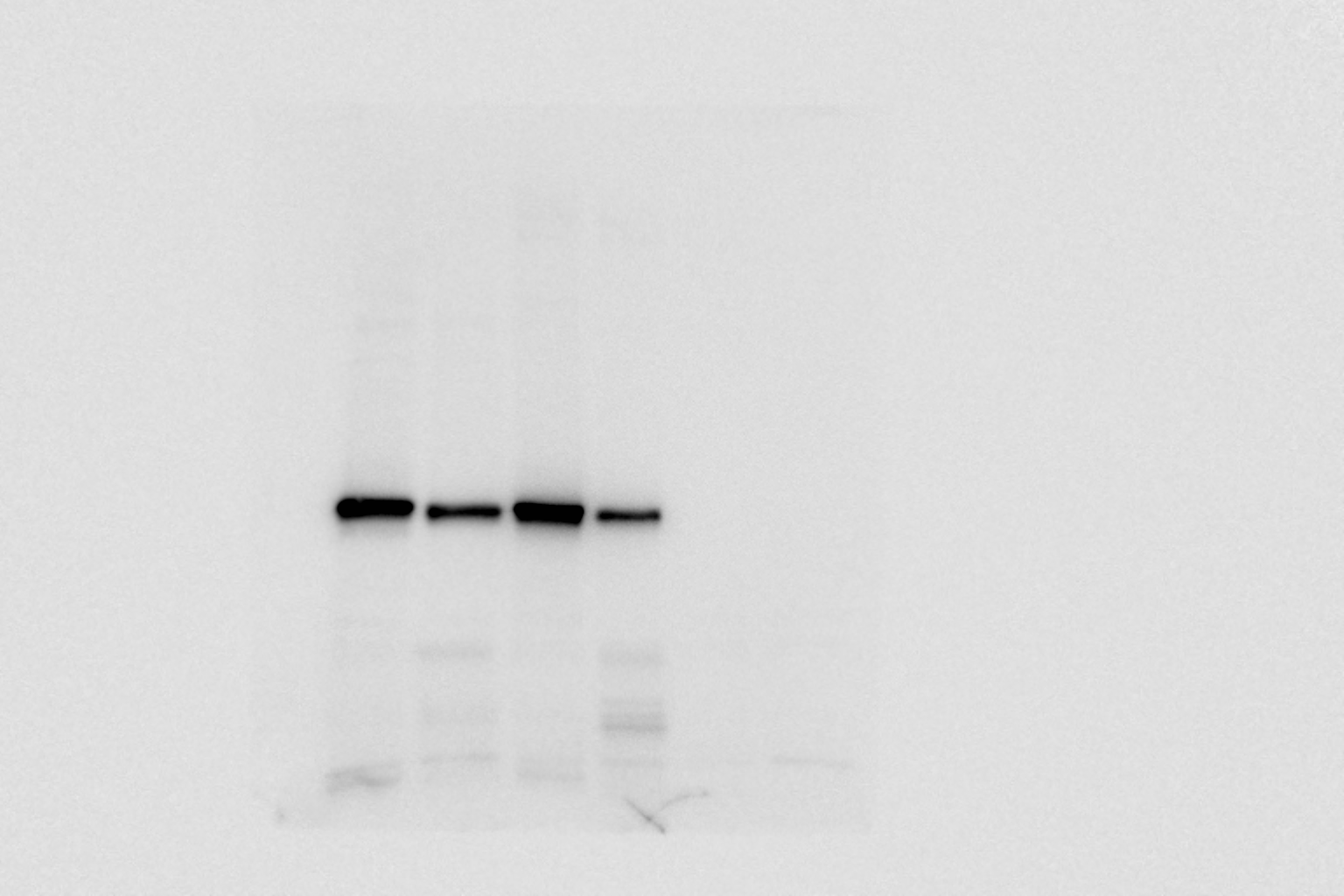

Supplement: Figure 1—figure supplement 2—source data 1. [file elife-93117-fig1-figsupp2-data1.zip › Fig. 1-Figure Supplement-2-Source data 1/Fig.1-Figure Supplement-2A-1-Source data 1.tif]

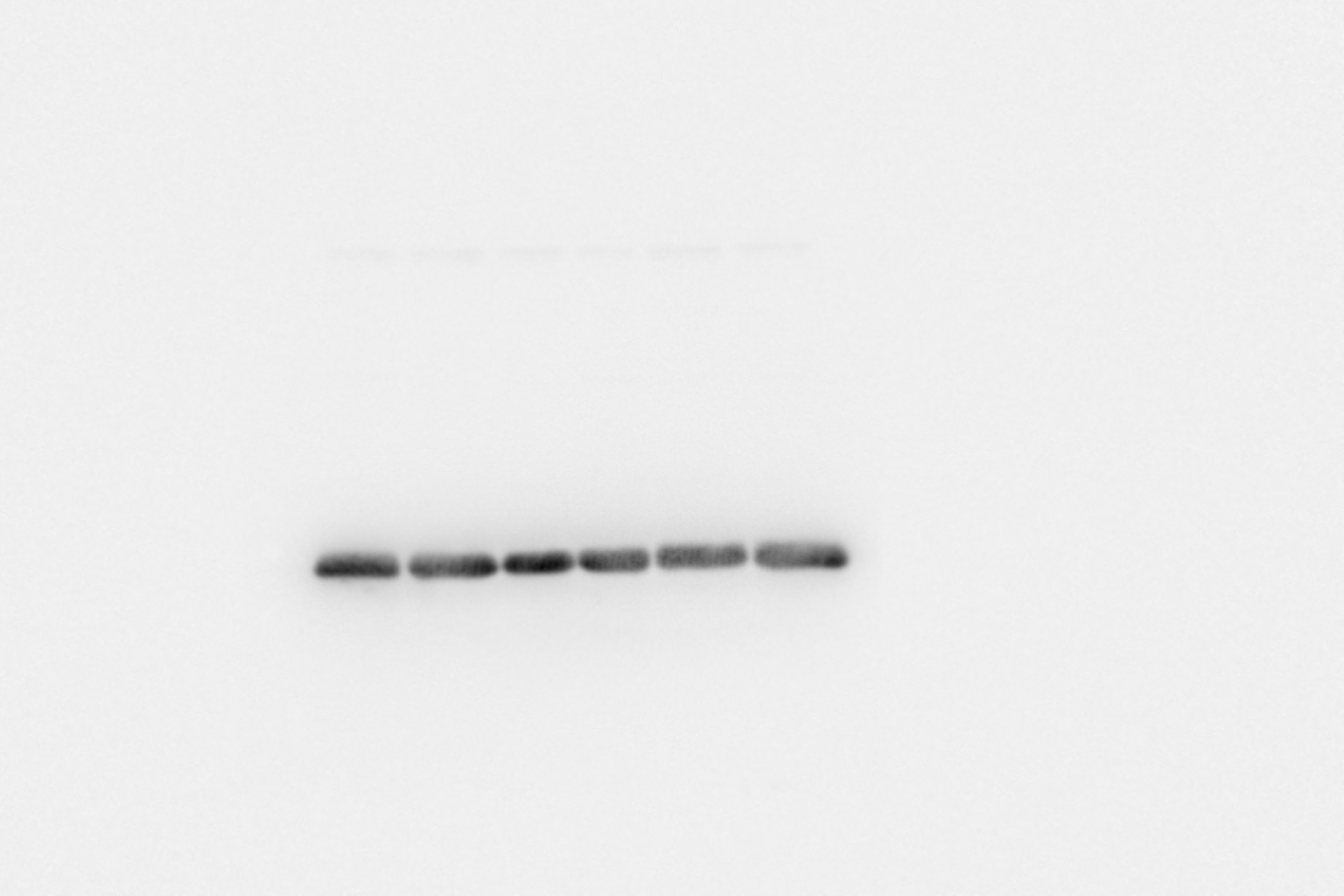

Supplement: Figure 1—figure supplement 2—source data 1. [file elife-93117-fig1-figsupp2-data1.zip › Fig. 1-Figure Supplement-2-Source data 1/Fig.1-Figure Supplement-2A-4-Source data 1.tif]

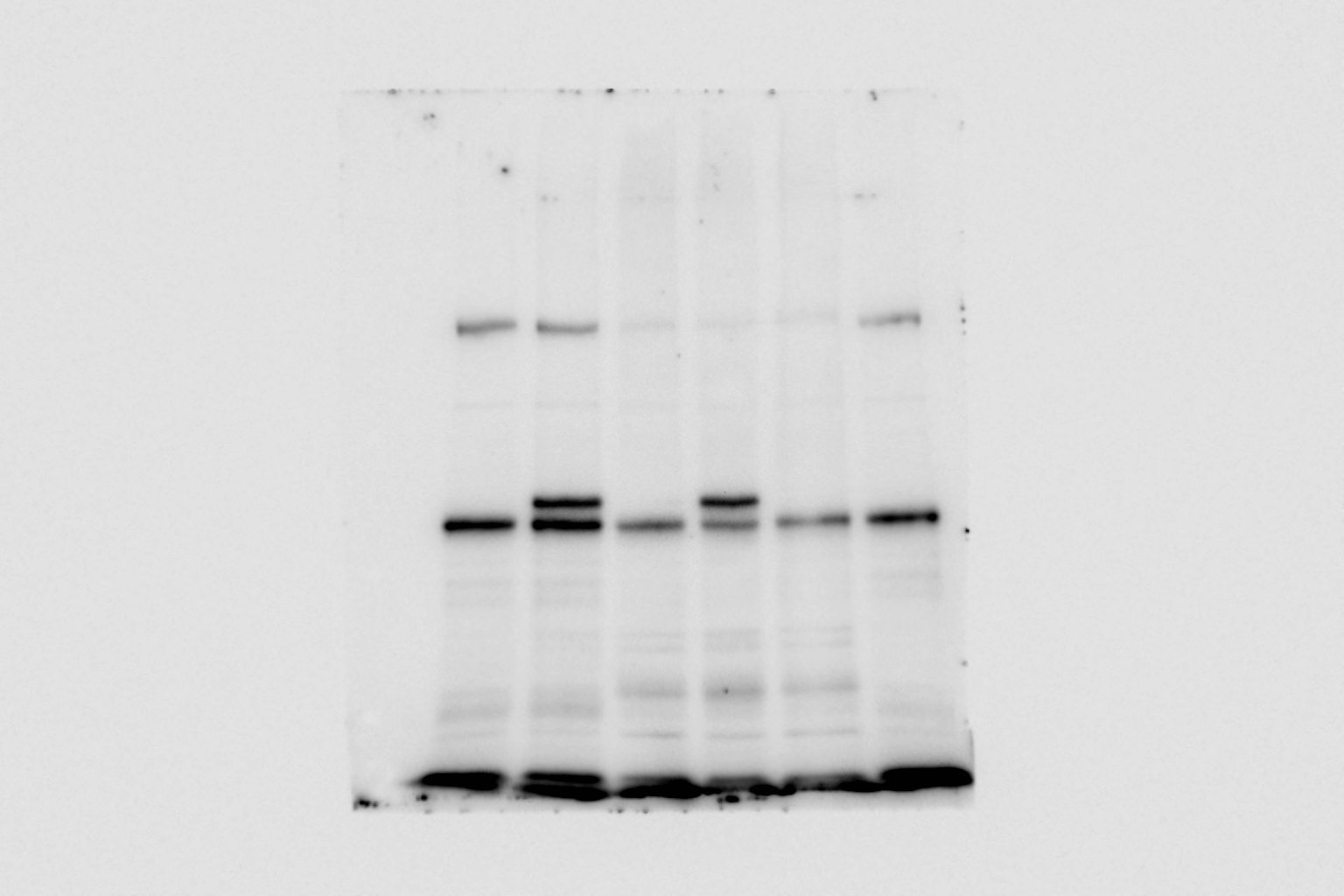

Supplement: Figure 1—figure supplement 2—source data 1. [file elife-93117-fig1-figsupp2-data1.zip › Fig. 1-Figure Supplement-2-Source data 1/Fig.1-Figure Supplement-2A-3-Source data 1.tif]

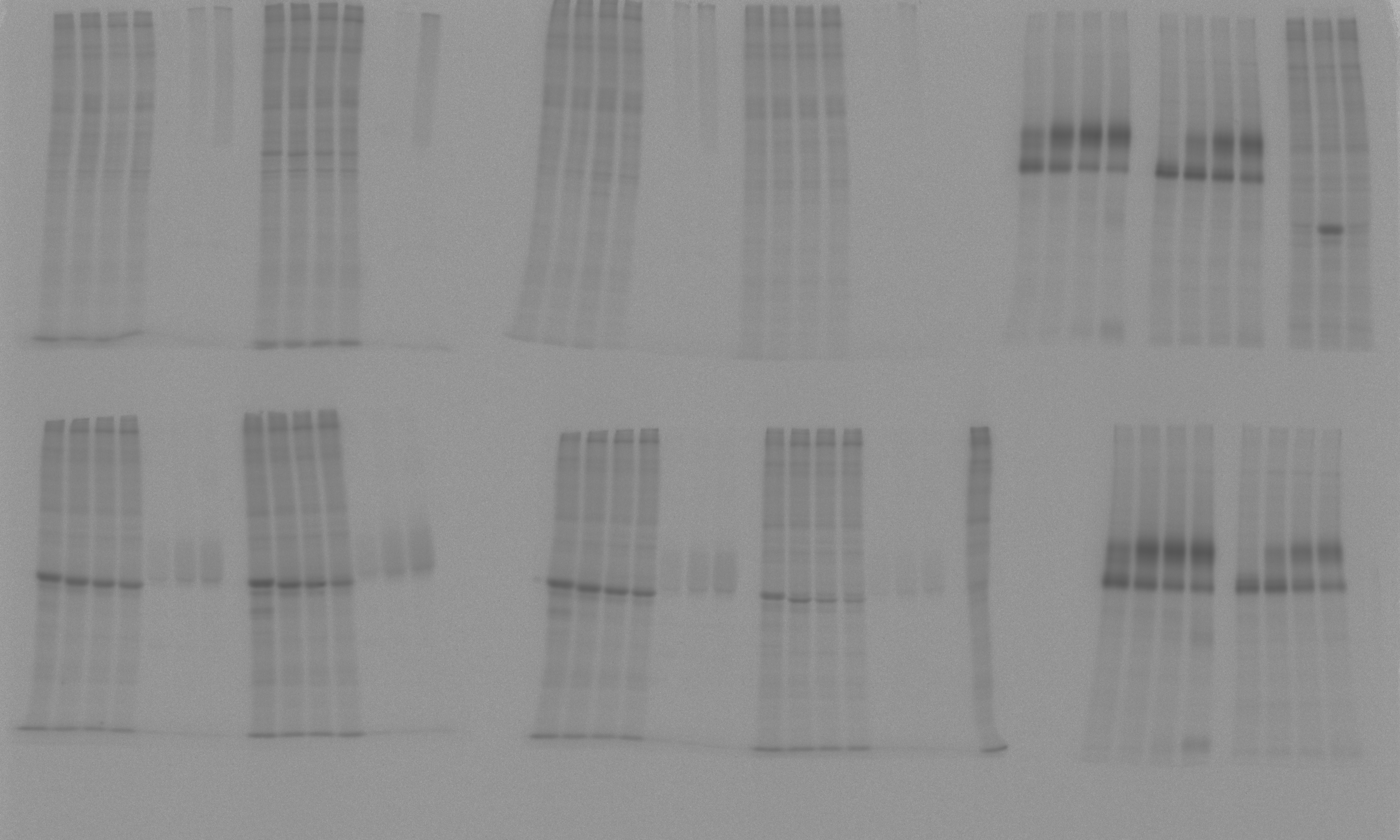

Supplement: Figure 1—figure supplement 2—source data 1. [file elife-93117-fig1-figsupp2-data1.zip › Fig. 1-Figure Supplement-2-Source data 1/Fig.1-Figure Supplement-2F-1-4-Source data 1.TIFF]

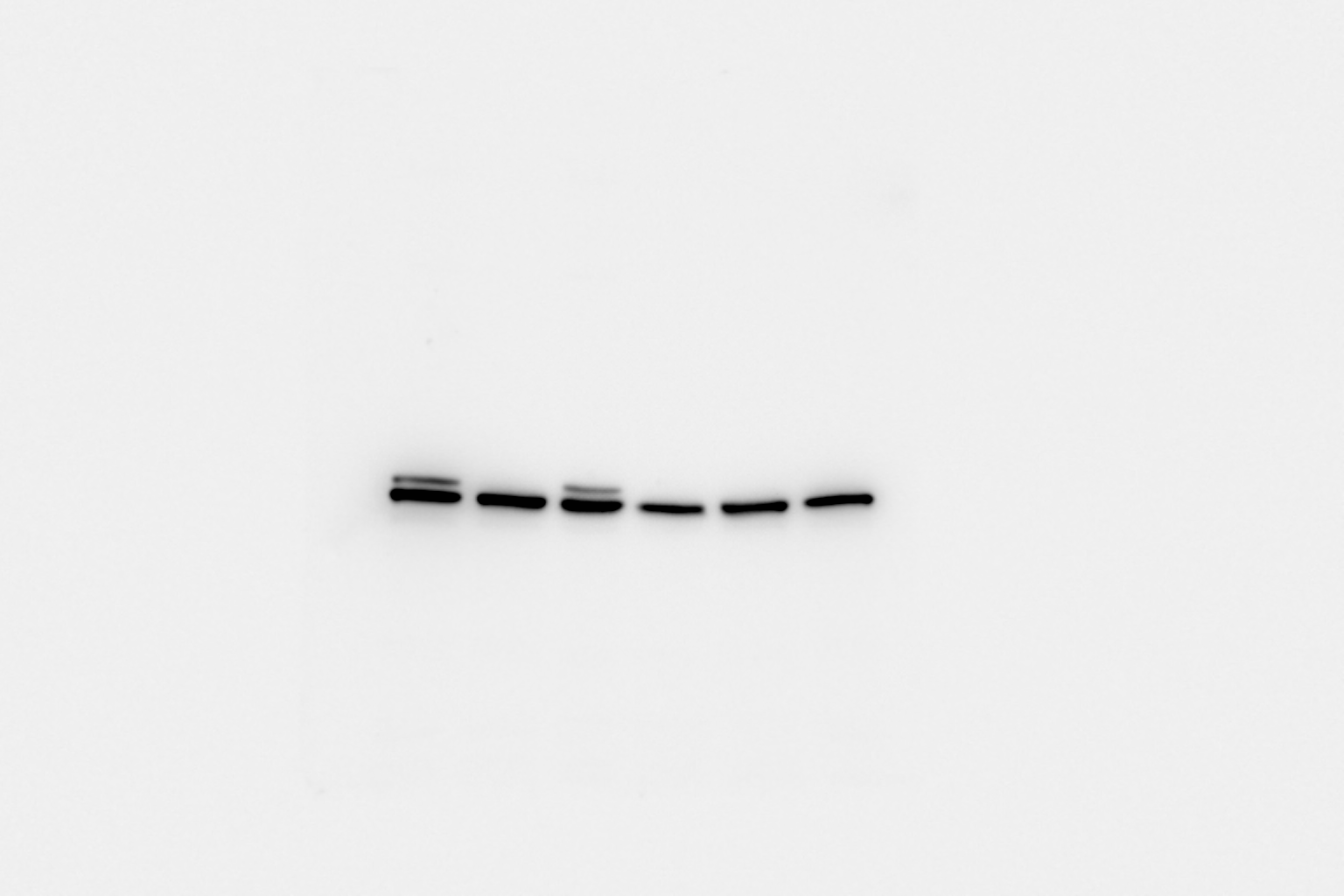

Supplement: Figure 1—figure supplement 2—source data 1. [file elife-93117-fig1-figsupp2-data1.zip › Fig. 1-Figure Supplement-2-Source data 1/Fig.1-Figure Supplement-2A-2-Source data 1.tif]

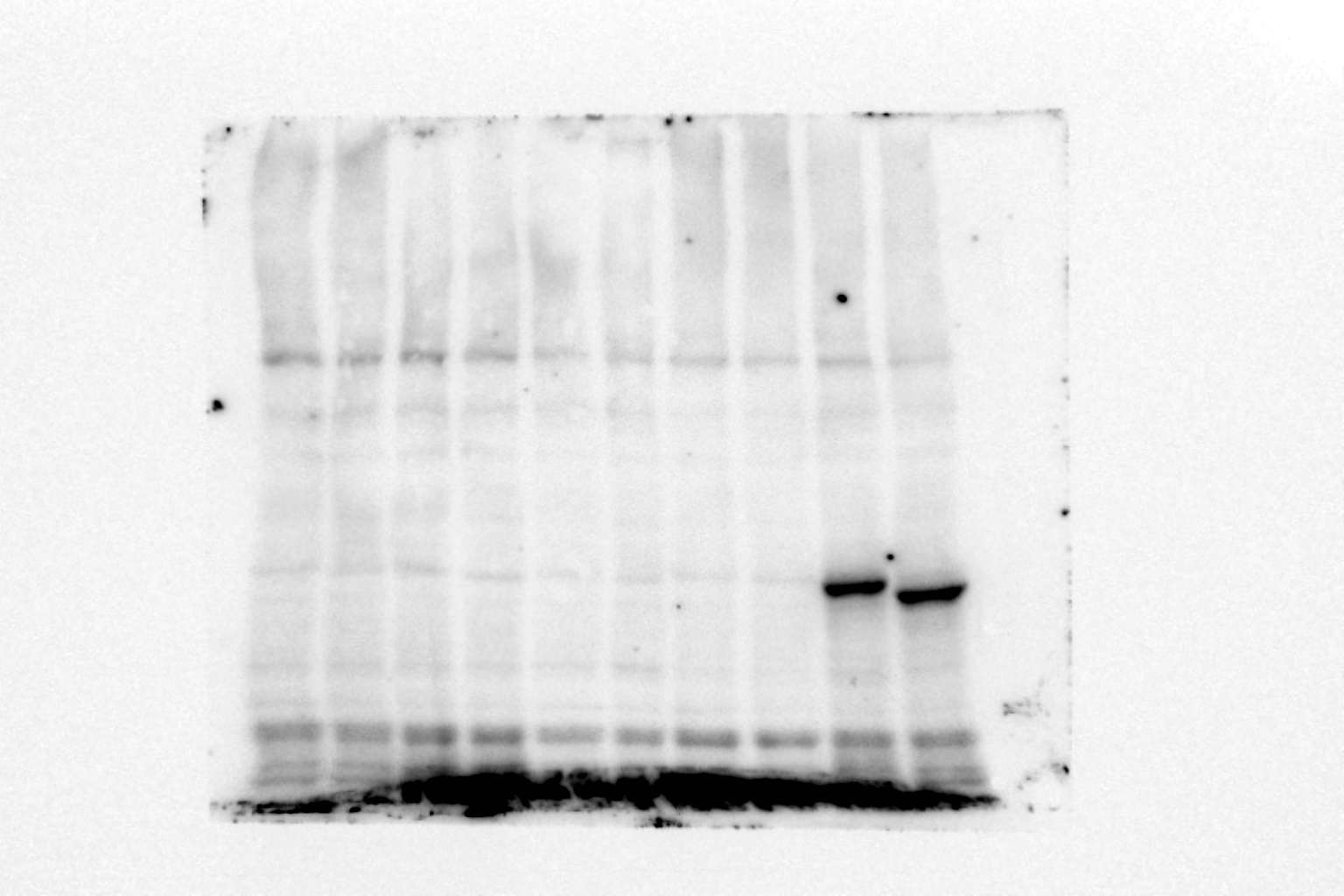

Supplement: Figure 1—figure supplement 2—source data 1. [file elife-93117-fig1-figsupp2-data1.zip › Fig. 1-Figure Supplement-2-Source data 1/Fig.1-Figure Supplement-2C-2-Source data 1.tif]

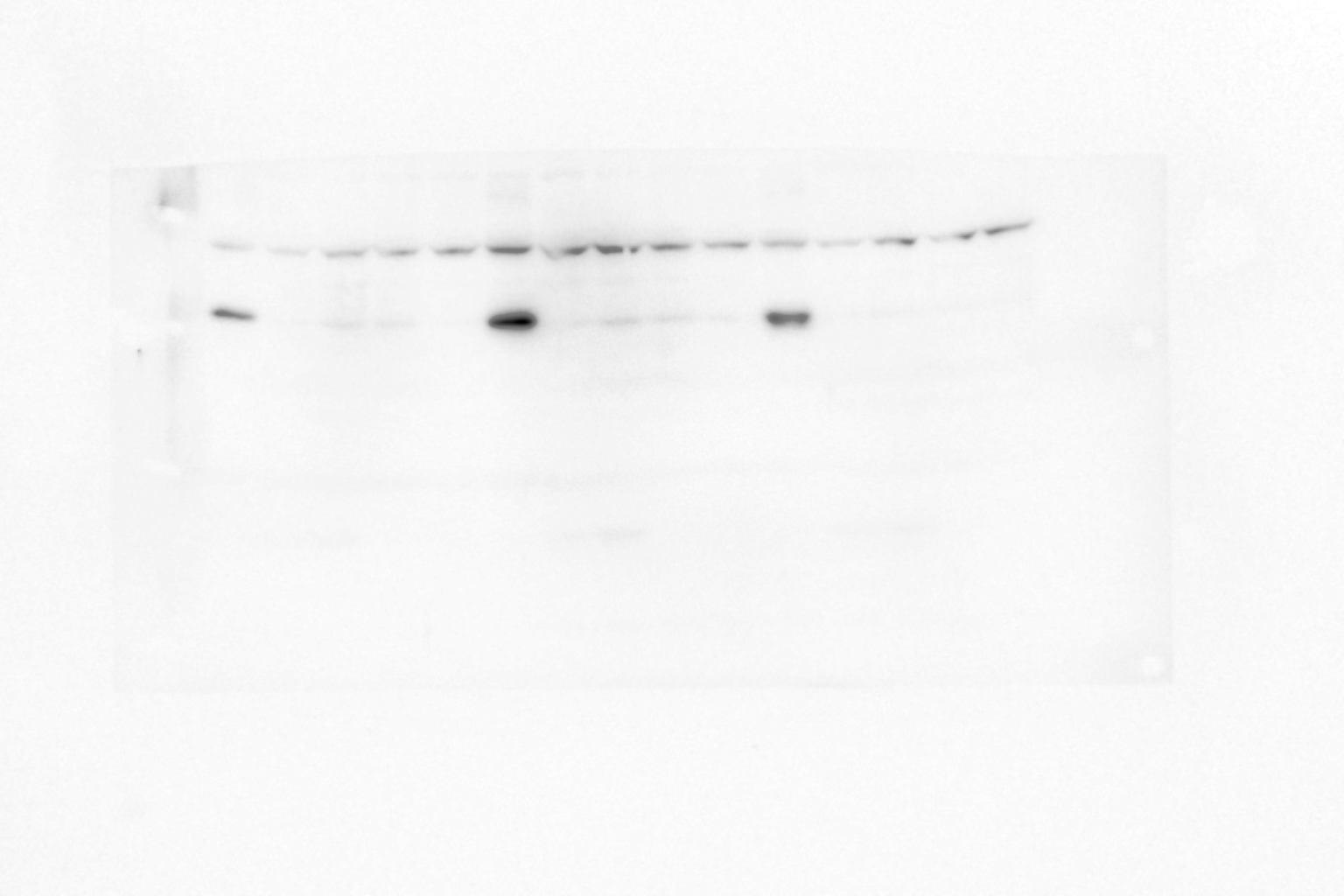

Supplement: Figure 1—figure supplement 2—source data 1. [file elife-93117-fig1-figsupp2-data1.zip › Fig. 1-Figure Supplement-2-Source data 1/Fig.1-Figure Supplement-2B-2-Source data 1.tif]

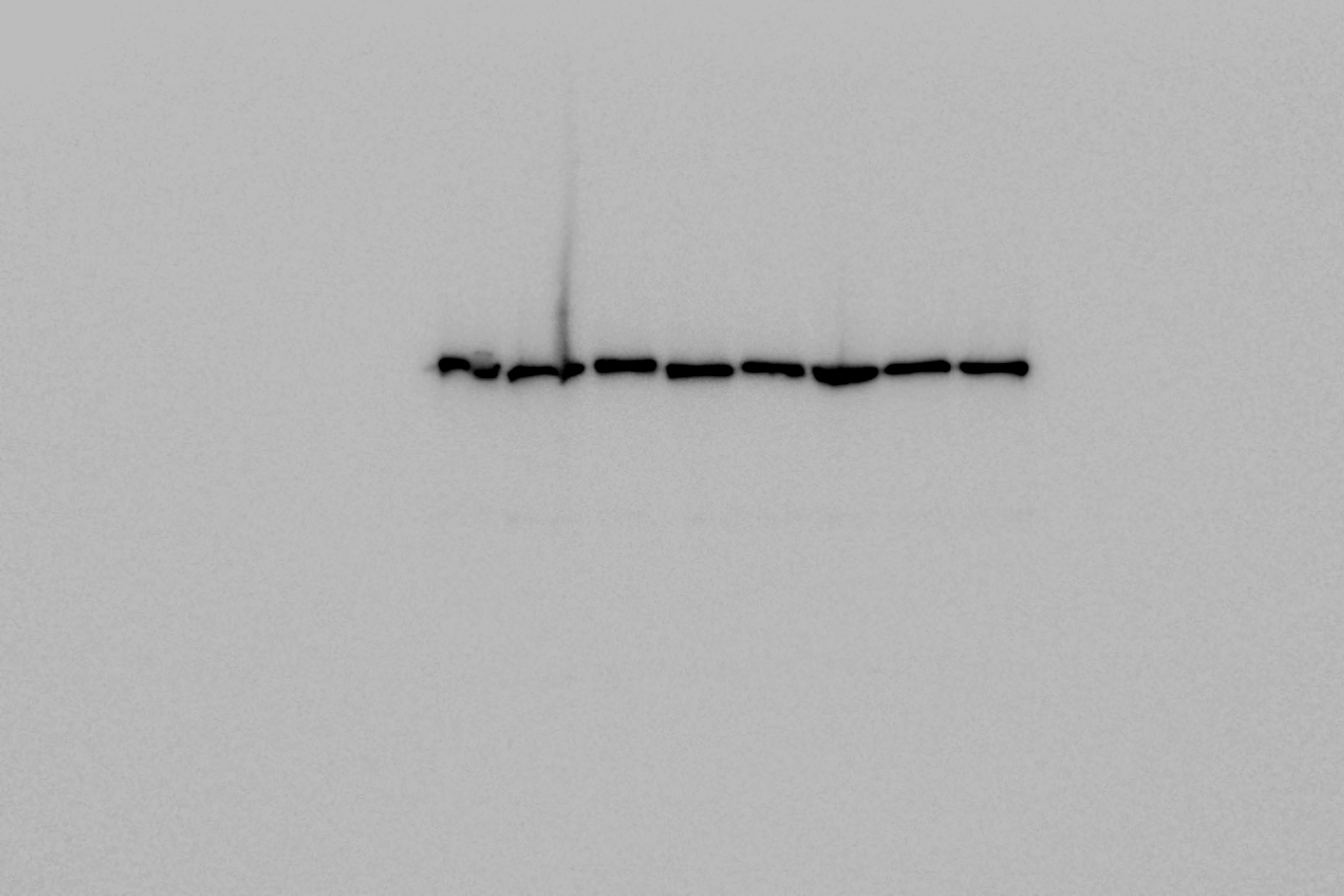

Supplement: Figure 1—figure supplement 2—source data 1. [file elife-93117-fig1-figsupp2-data1.zip › Fig. 1-Figure Supplement-2-Source data 1/Fig.1-Figure Supplement-2C-1-Source data 1.jpg]

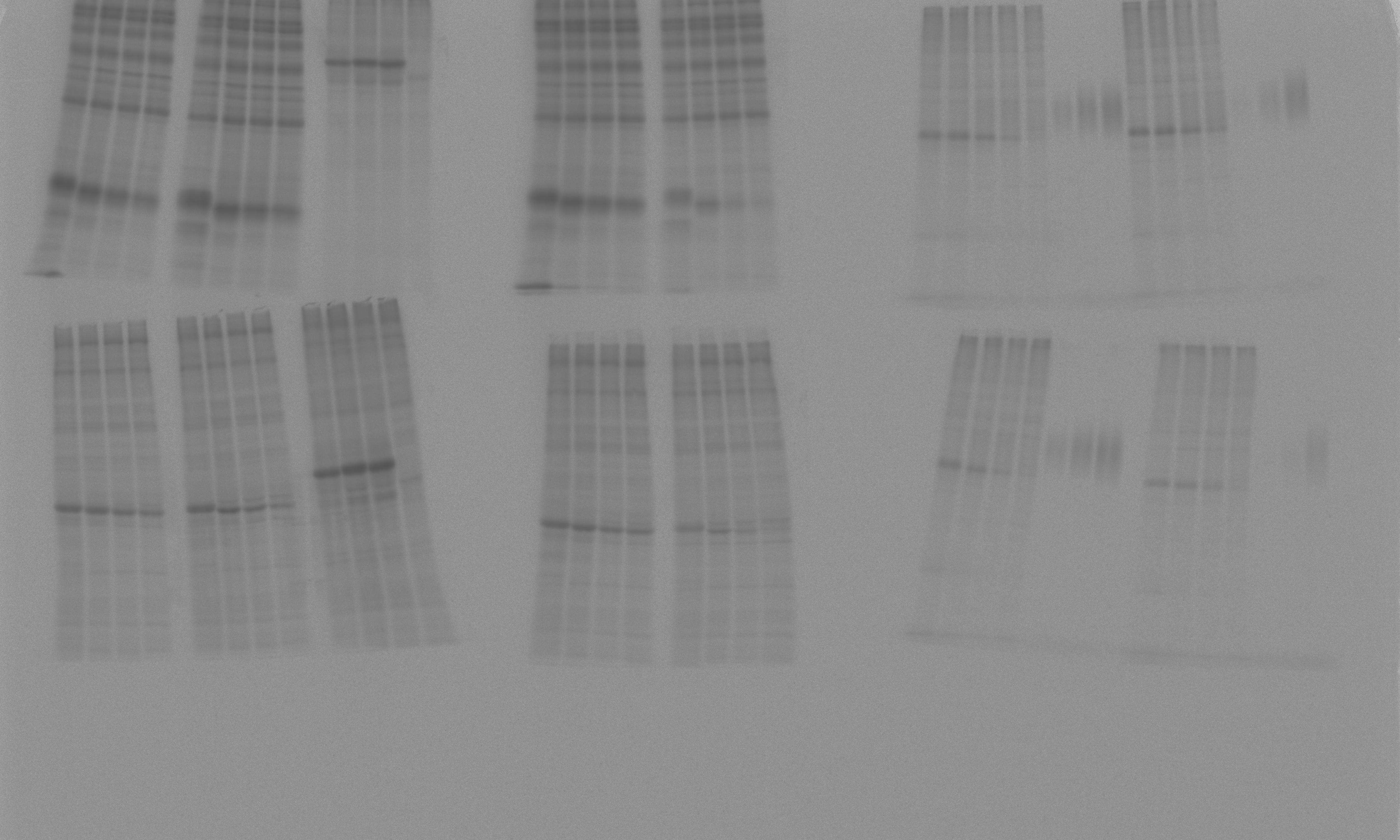

Supplement: Figure 1—figure supplement 2—source data 1. [file elife-93117-fig1-figsupp2-data1.zip › Fig. 1-Figure Supplement-2-Source data 1/Fig.1-Figure Supplement-2E-1-4-Source data 1.TIFF]

Fig. 1-Figure Supplement 6 Original gels corresponding to Fig1-Fig. Sup.6A  
Fig. Sup.6A

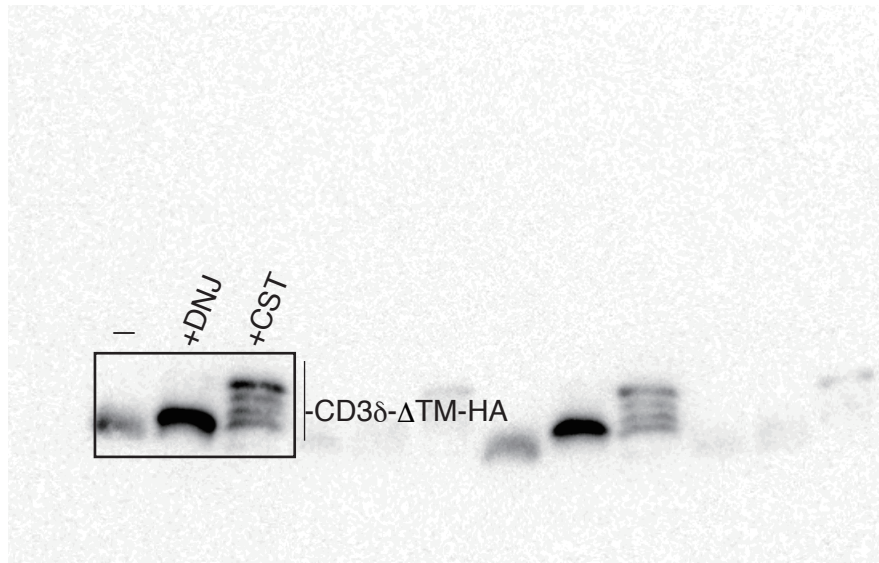

Fig. Sup.3F

Supplement: Figure 1—figure supplement 6—source data 2. [file elife-93117-fig1-figsupp6-data2.pdf]

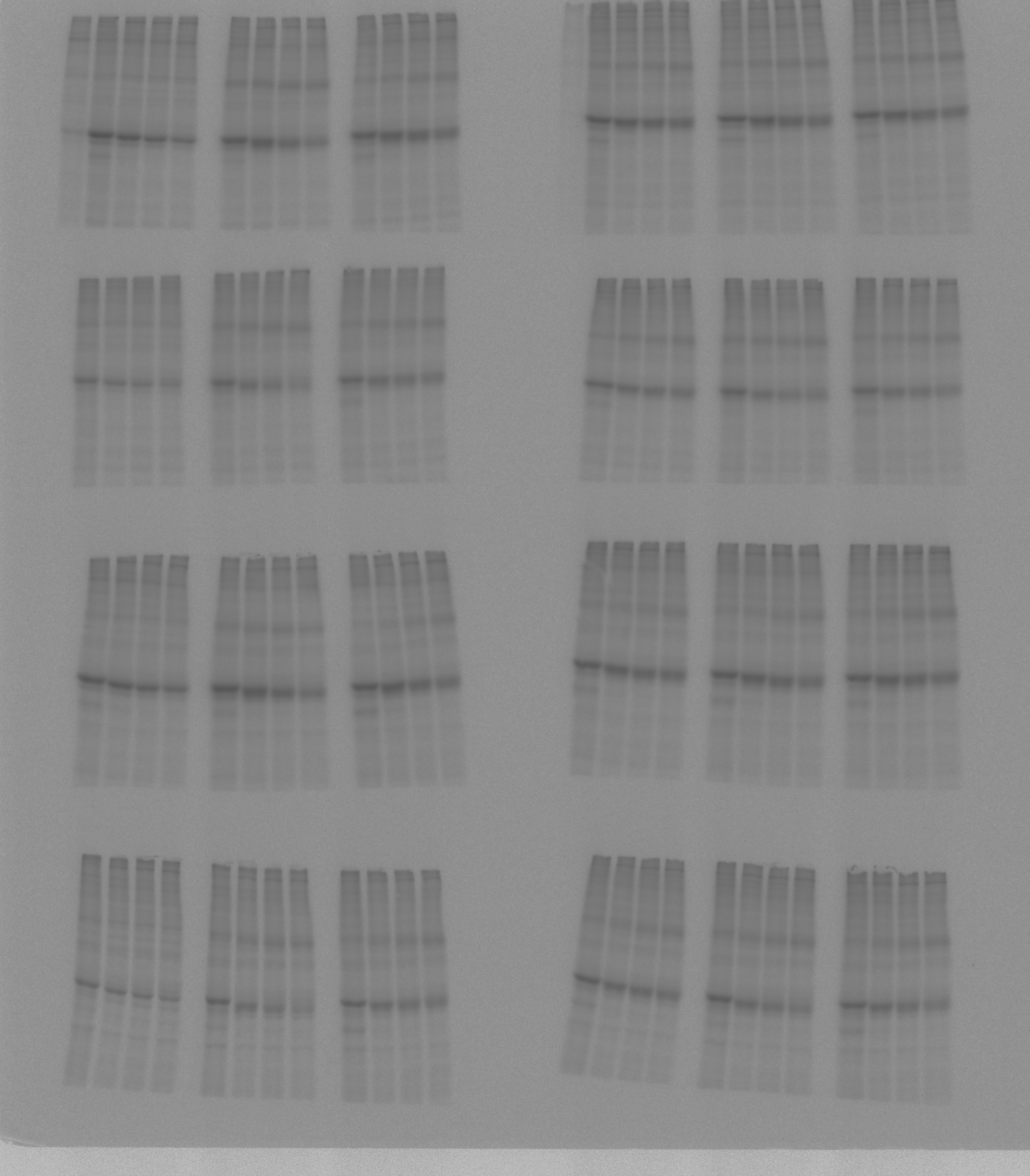

Supplement: Figure 2—source data 1. [file elife-93117-fig2-data1.zip › Fig. 2-Source data 1/Fig.2E-1-2,4-5-Source data 1.TIFF]

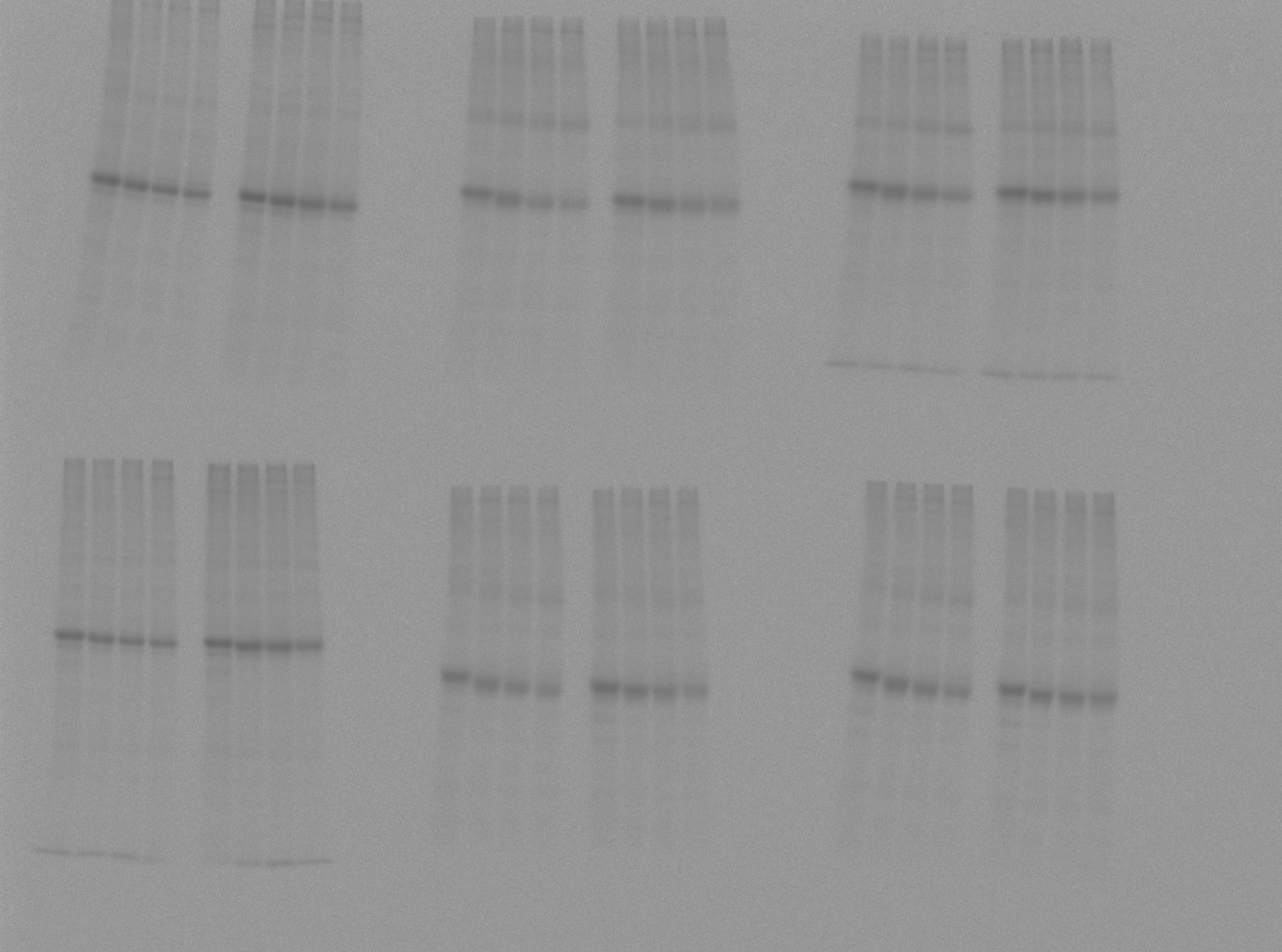

Supplement: Figure 2—source data 1. [file elife-93117-fig2-data1.zip › Fig. 2-Source data 1/Fig.2D-1-2-Source data 1.TIFF]

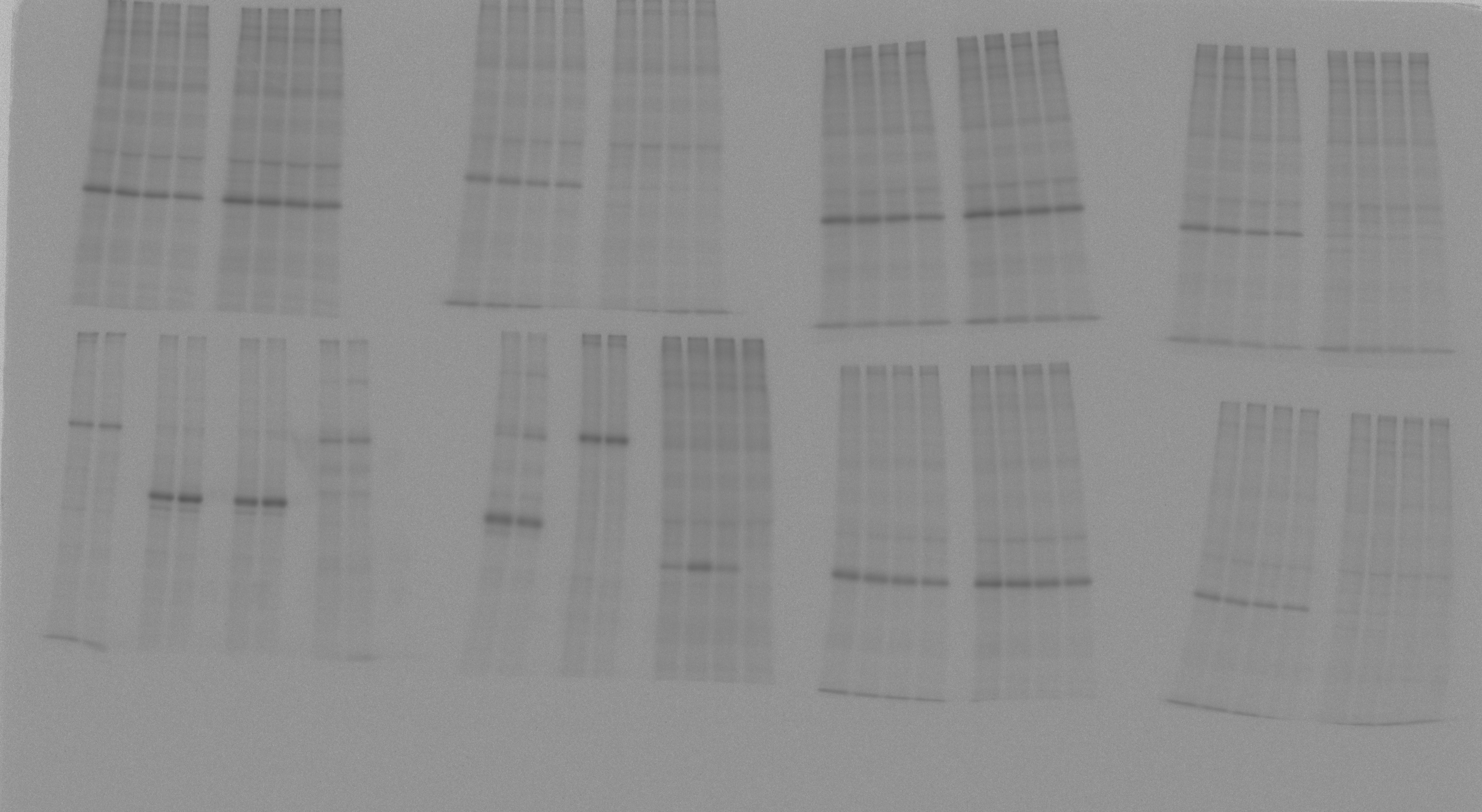

Supplement: Figure 2—source data 1. [file elife-93117-fig2-data1.zip › Fig. 2-Source data 1/Fig.2C-1-3-Source data 1.TIFF]

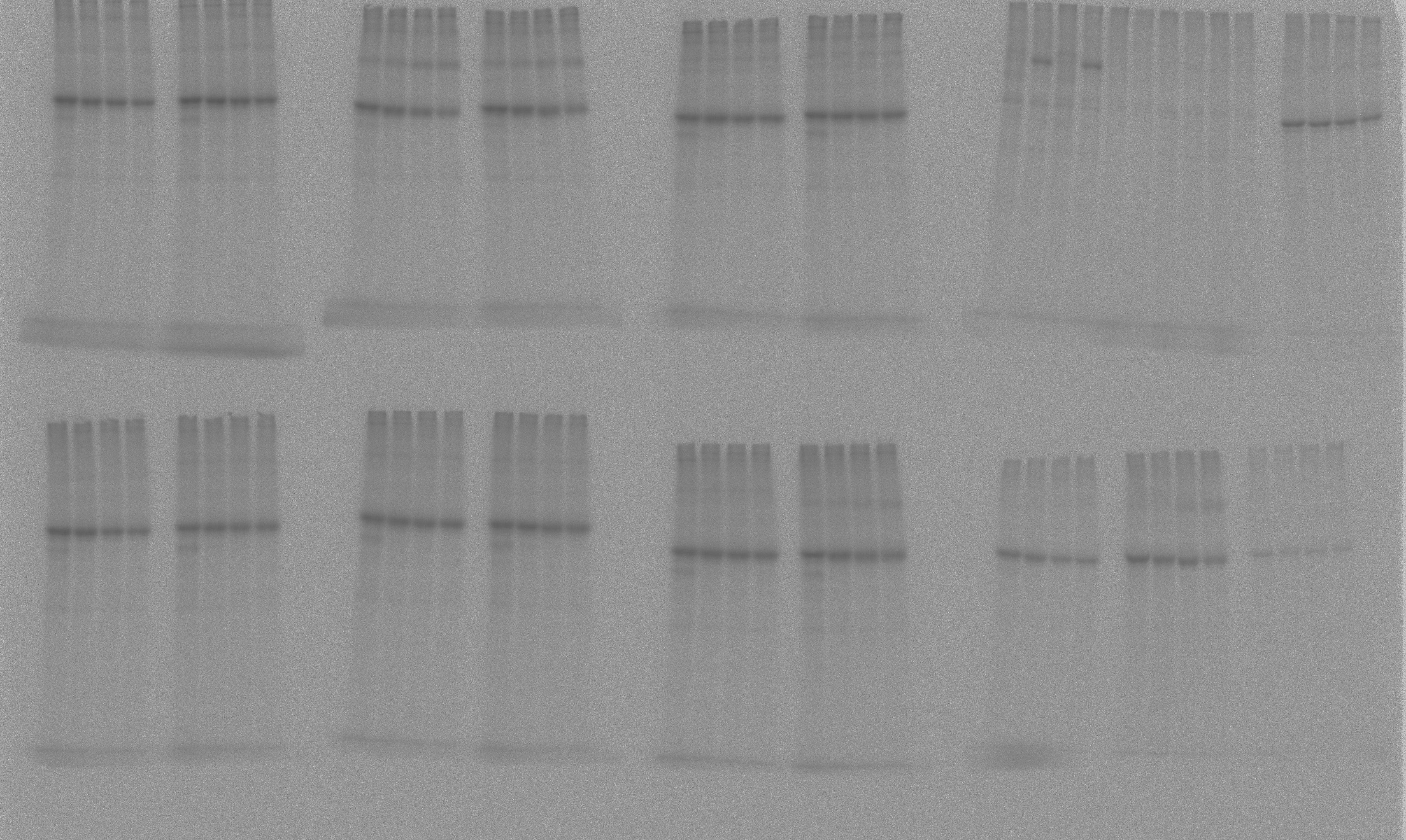

Supplement: Figure 2—source data 1. [file elife-93117-fig2-data1.zip › Fig. 2-Source data 1/Fig.2D-3-4-Source data 1.TIFF]

Fig. 2E Source data 2 Original gels corresponding to Fig. 2E.

Fig. 2E

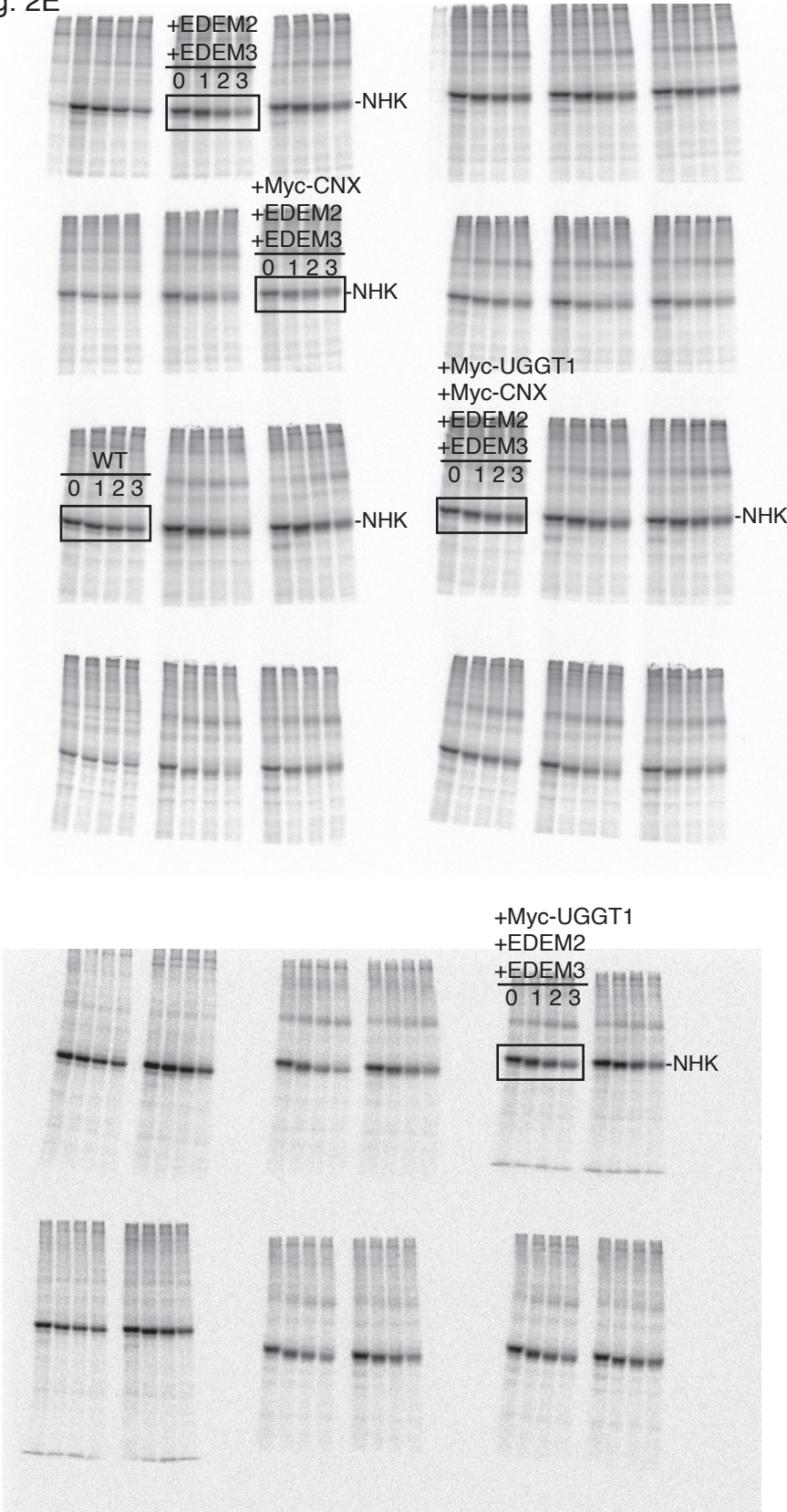

Supplement: Figure 2—source data 2. [file elife-93117-fig2-data2.zip › Fig. 2-Source data 2/Fig. 2E-Source data 2.pdf]

Fig. 2D Source data 2 Original gels corresponding to Fig2D

Fig. 2D

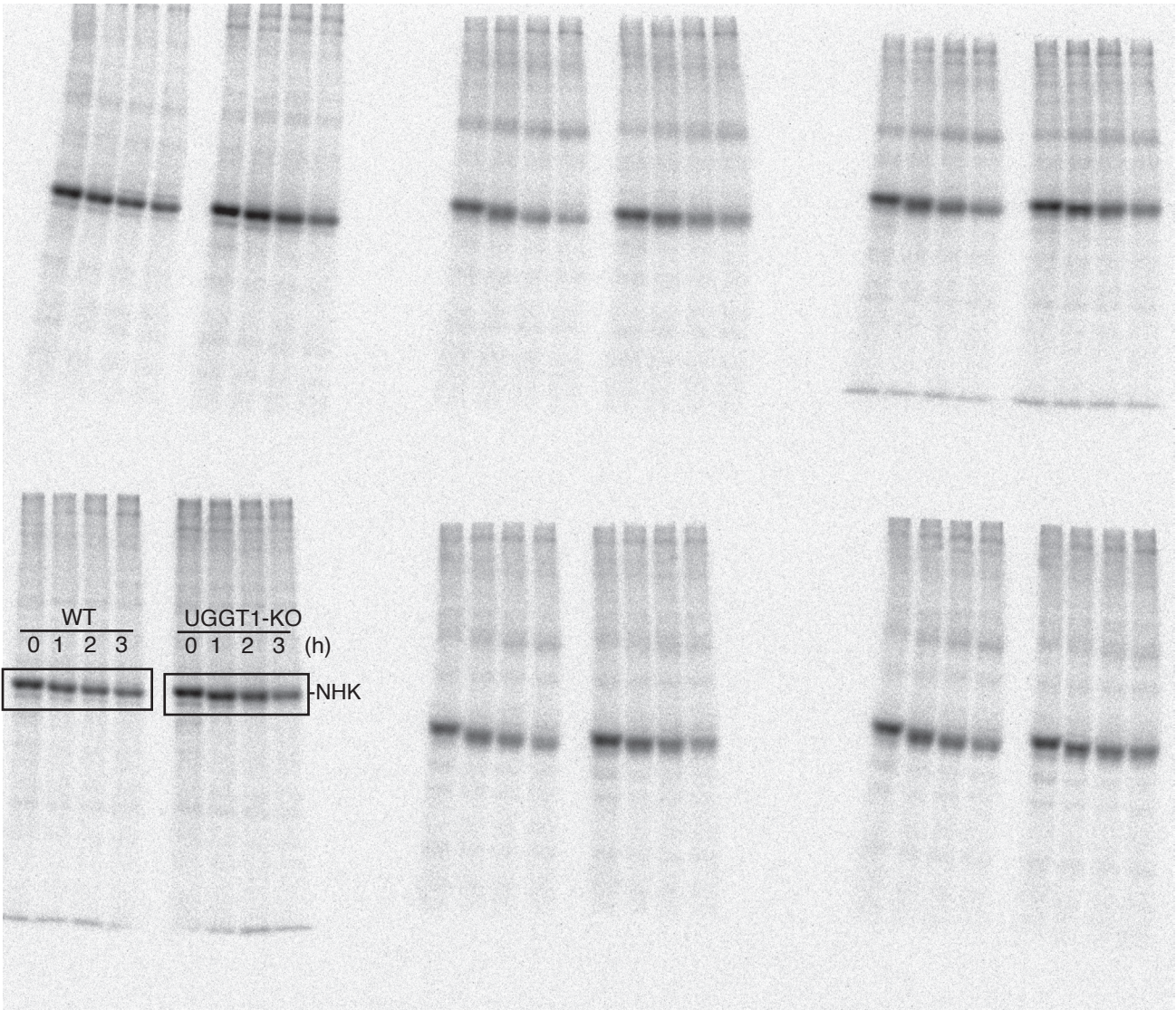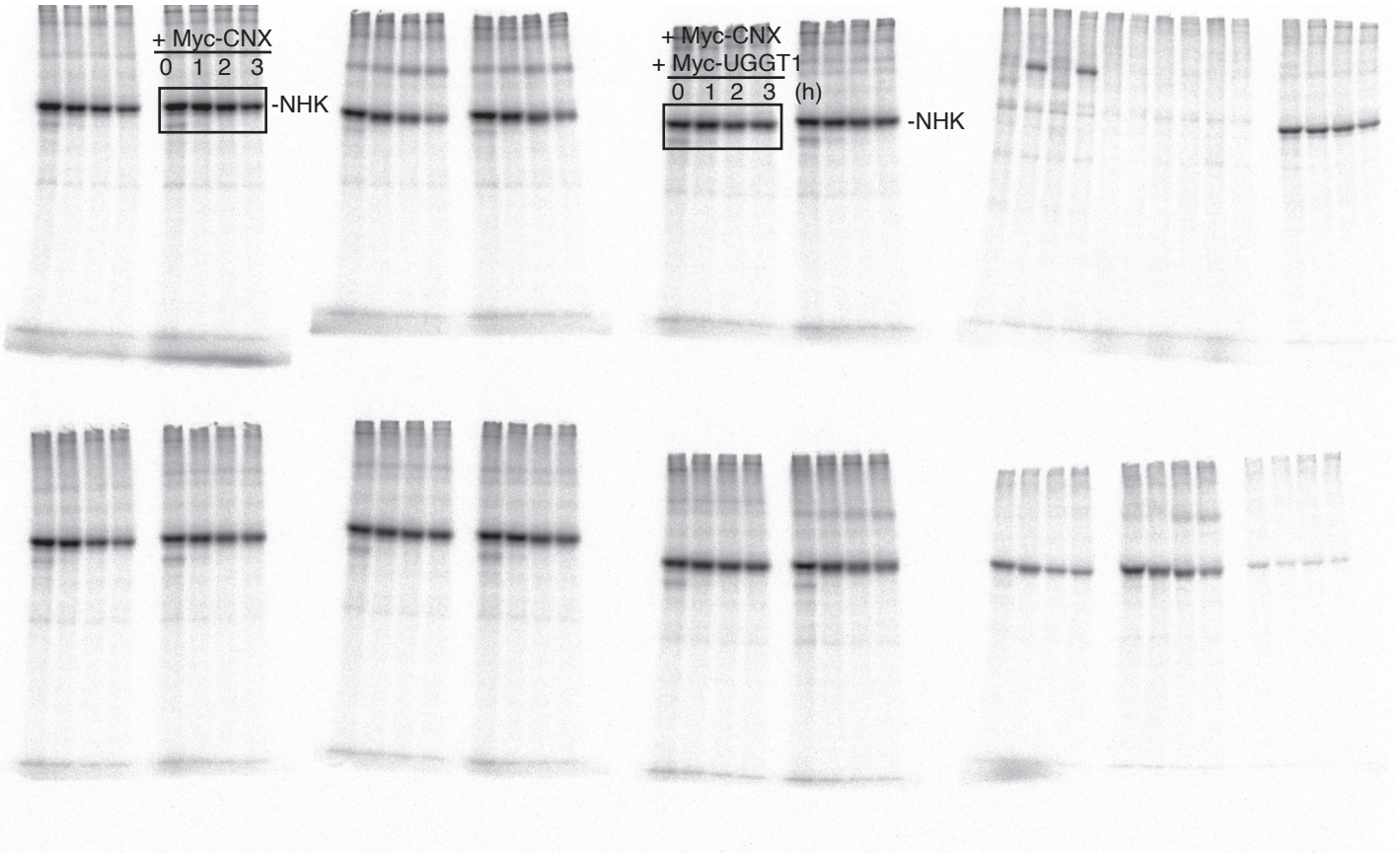

Supplement: Figure 2—source data 2. [file elife-93117-fig2-data2.zip › Fig. 2-Source data 2/Fig. 2D-Source data 2.pdf]

Fig. 2A Source data 2 Original gels corresponding to Fig2A

Fig. 2A

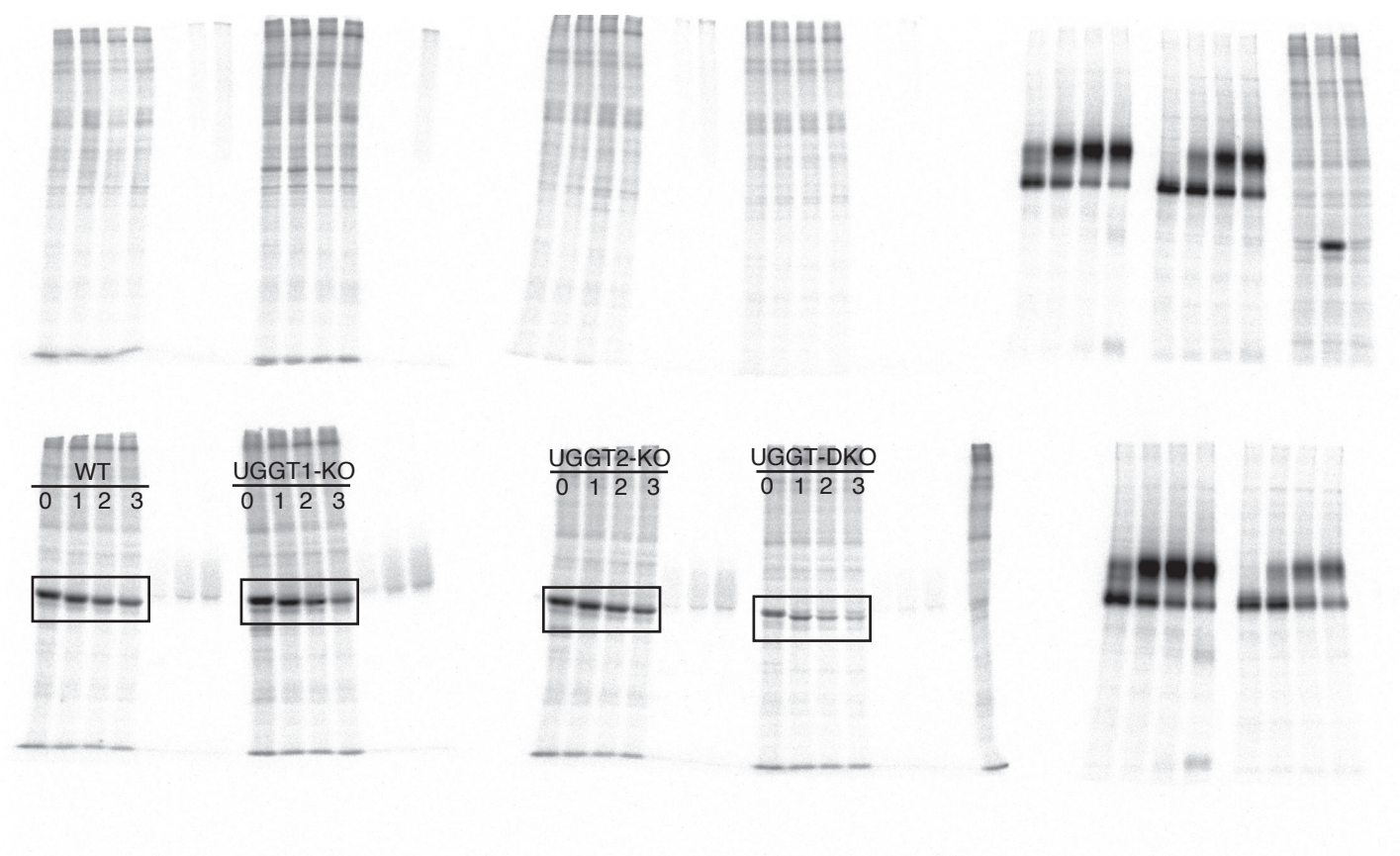

Supplement: Figure 2—source data 2. [file elife-93117-fig2-data2.zip › Fig. 2-Source data 2/Fig. 2A-Source data 2.pdf]

Fig. 2B Source data 2 Original gels corresponding to Fig2B

Fig. 2B

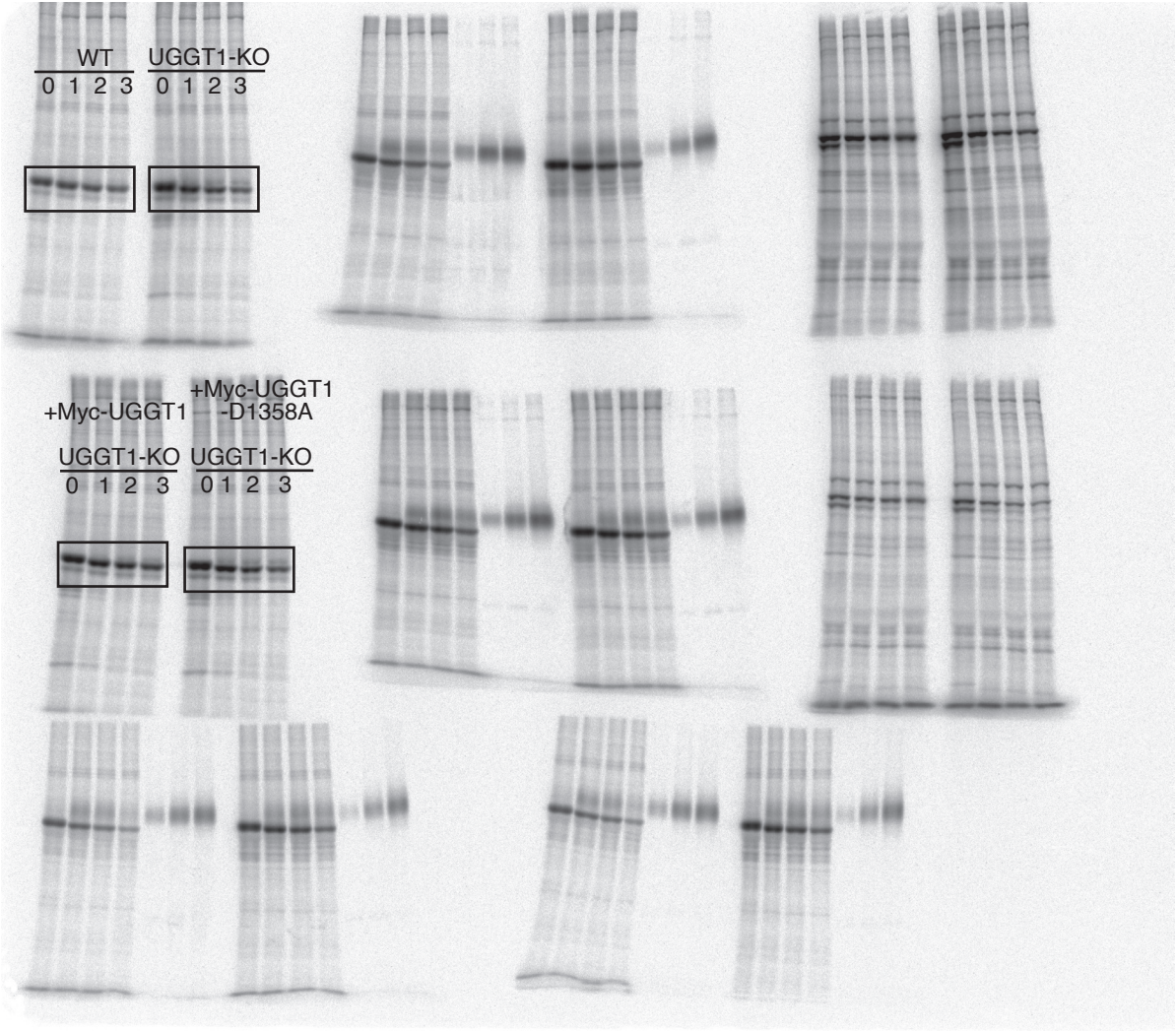

Supplement: Figure 2—source data 2. [file elife-93117-fig2-data2.zip › Fig. 2-Source data 2/Fig. 2B-Source data 2.pdf]

Fig. 2C Source data 2 Original gels corresponding to Fig2C

Fig. 2C

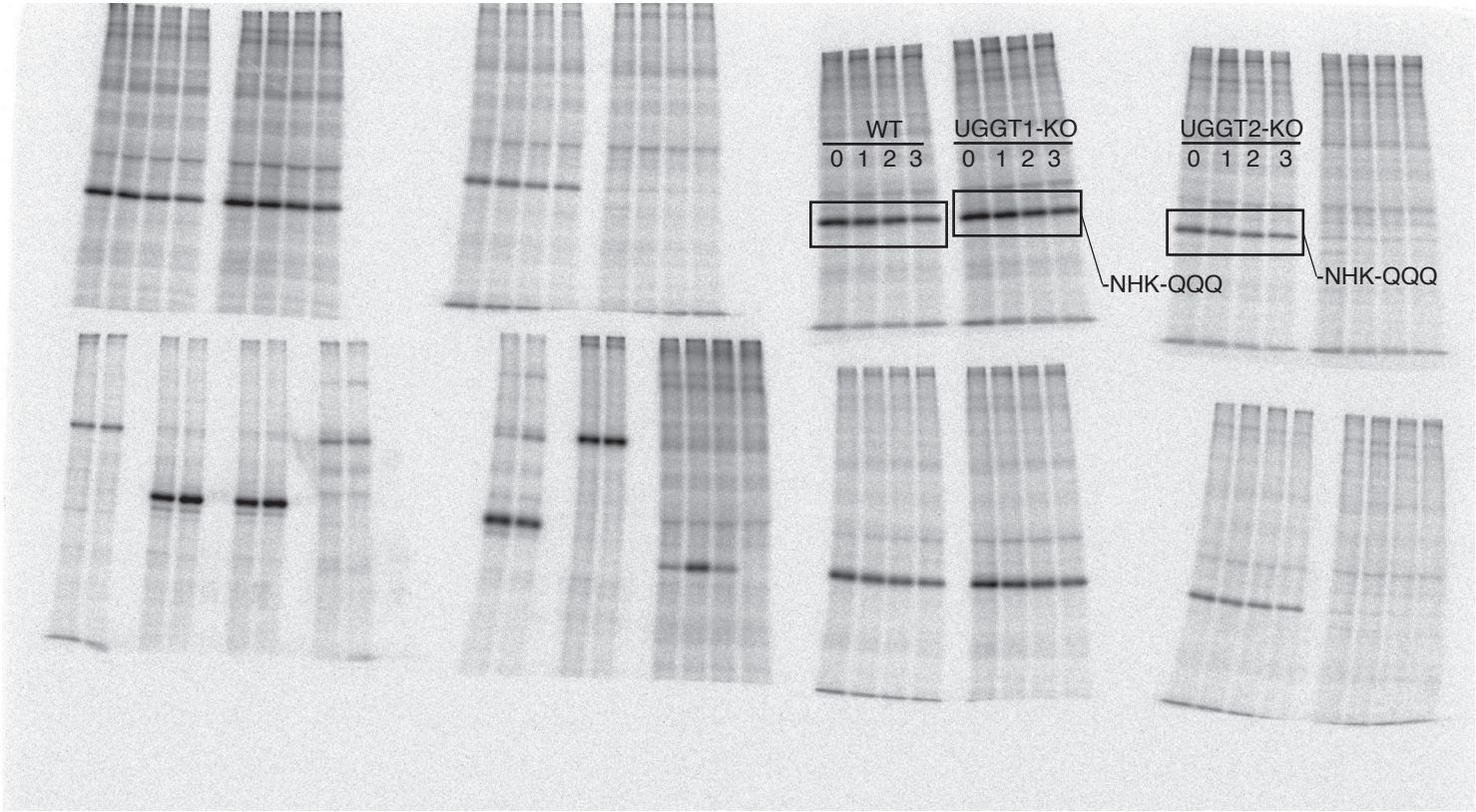

Supplement: Figure 2—source data 2. [file elife-93117-fig2-data2.zip › Fig. 2-Source data 2/Fig. 2C-Source data 2.pdf]

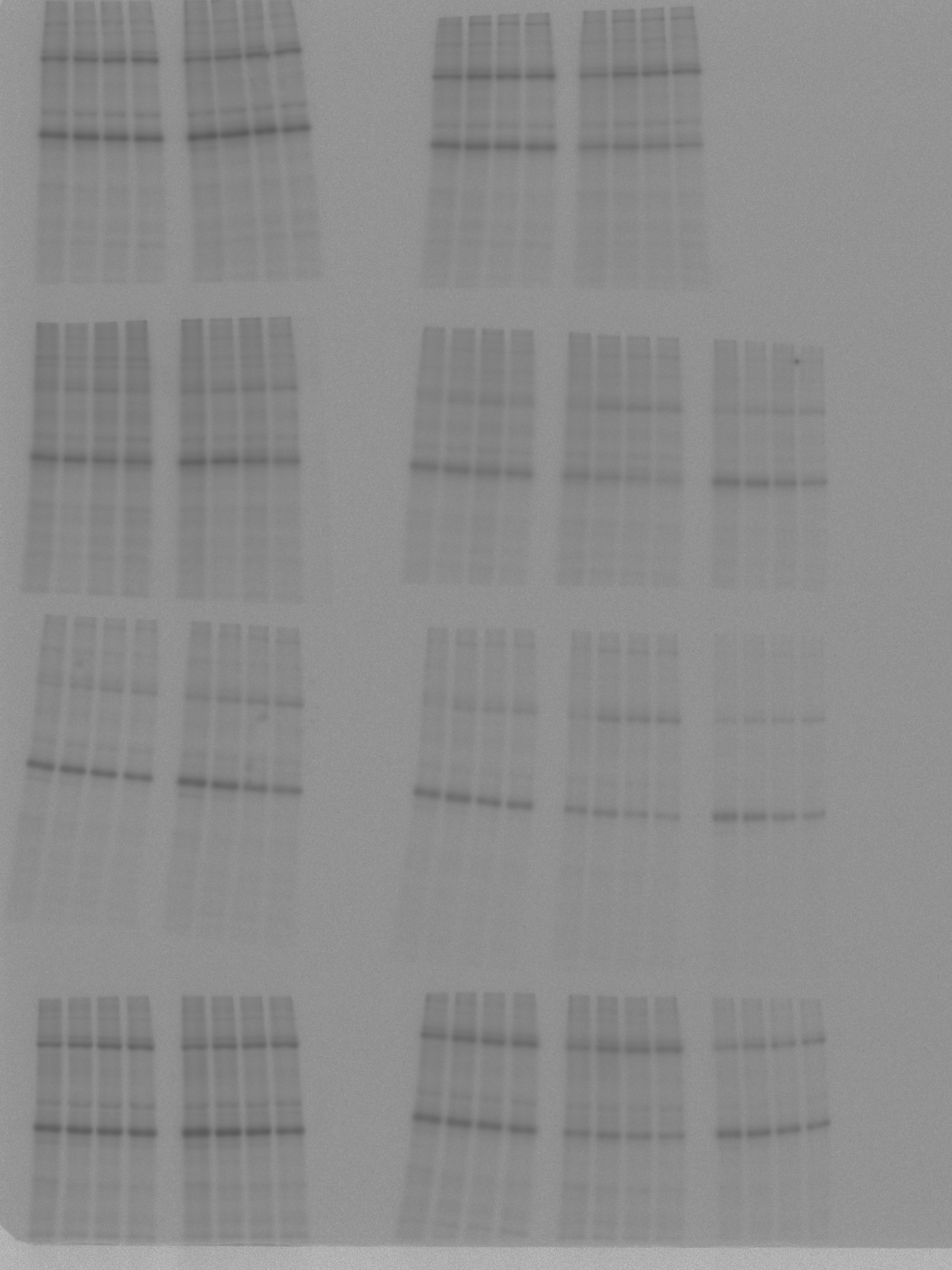

Supplement: Figure 3—source data 1. [file elife-93117-fig3-data1.zip › Fig. 3-Source data 1/Fig.3D-3-6-Source data 1.TIFF]

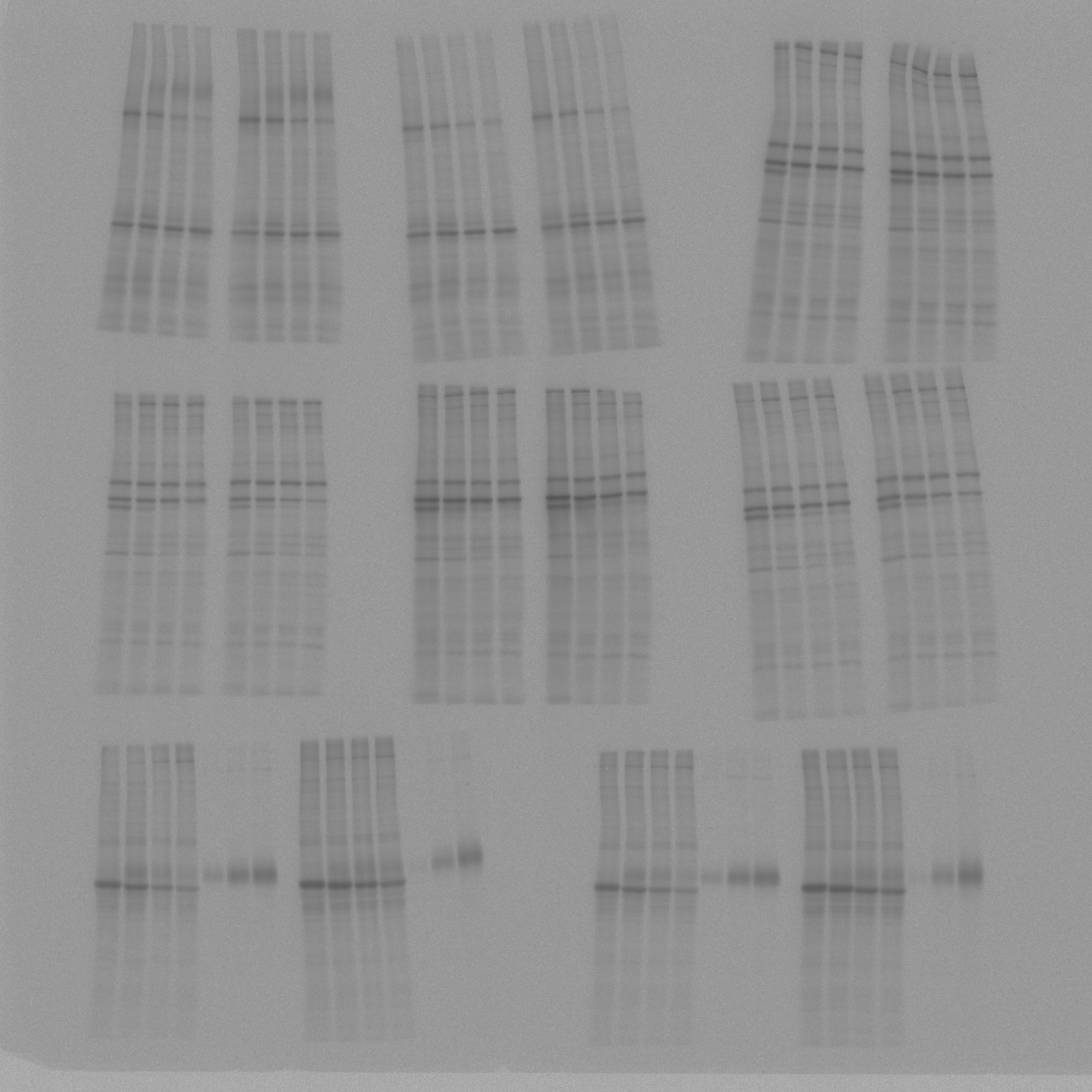

Supplement: Figure 3—source data 1. [file elife-93117-fig3-data1.zip › Fig. 3-Source data 1/Fig.3F-1-4-Source data 1.TIFF]

Fig. 3A-C Source data 2 Original membranes corresponding to Fig3A-C.

Fig. 3A

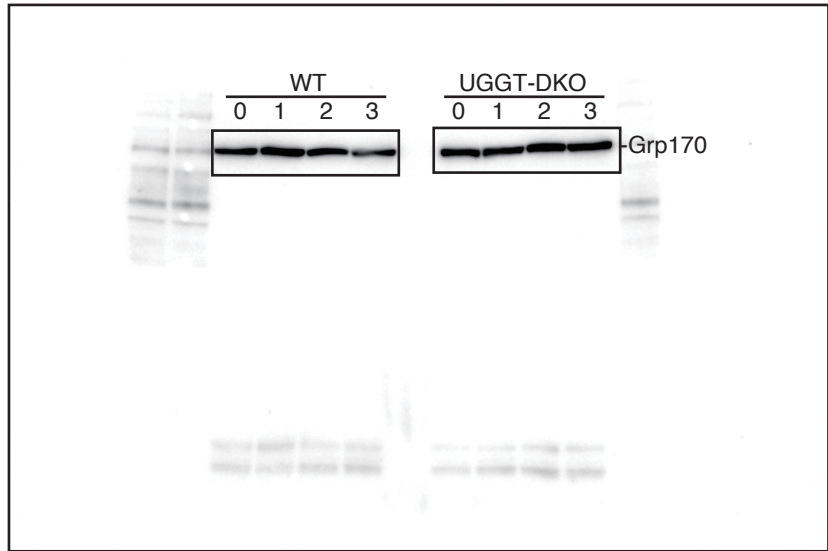

Fig. 3B

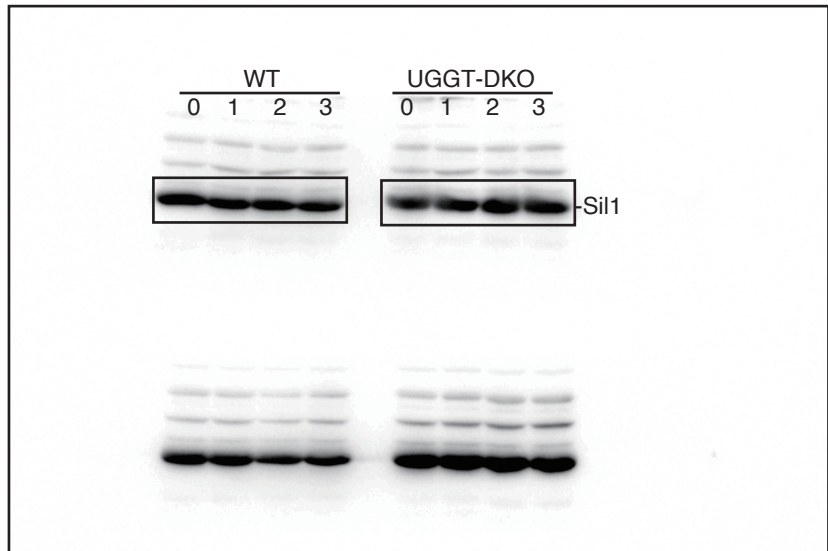

Fig. 3C

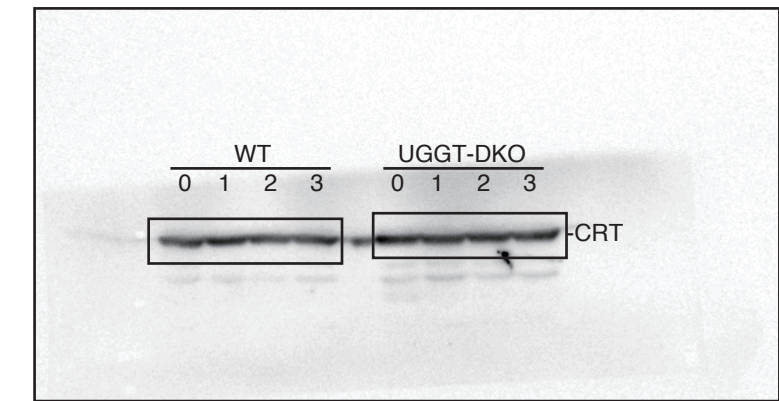

Supplement: Figure 3—source data 2. [file elife-93117-fig3-data2.zip › Fig. 3-Source data 2/Fig. 3A-C-Source data 2.pdf]

Fig. 3F Source data 2 Original gels corresponding to Fig. 3F

Fig. 3F

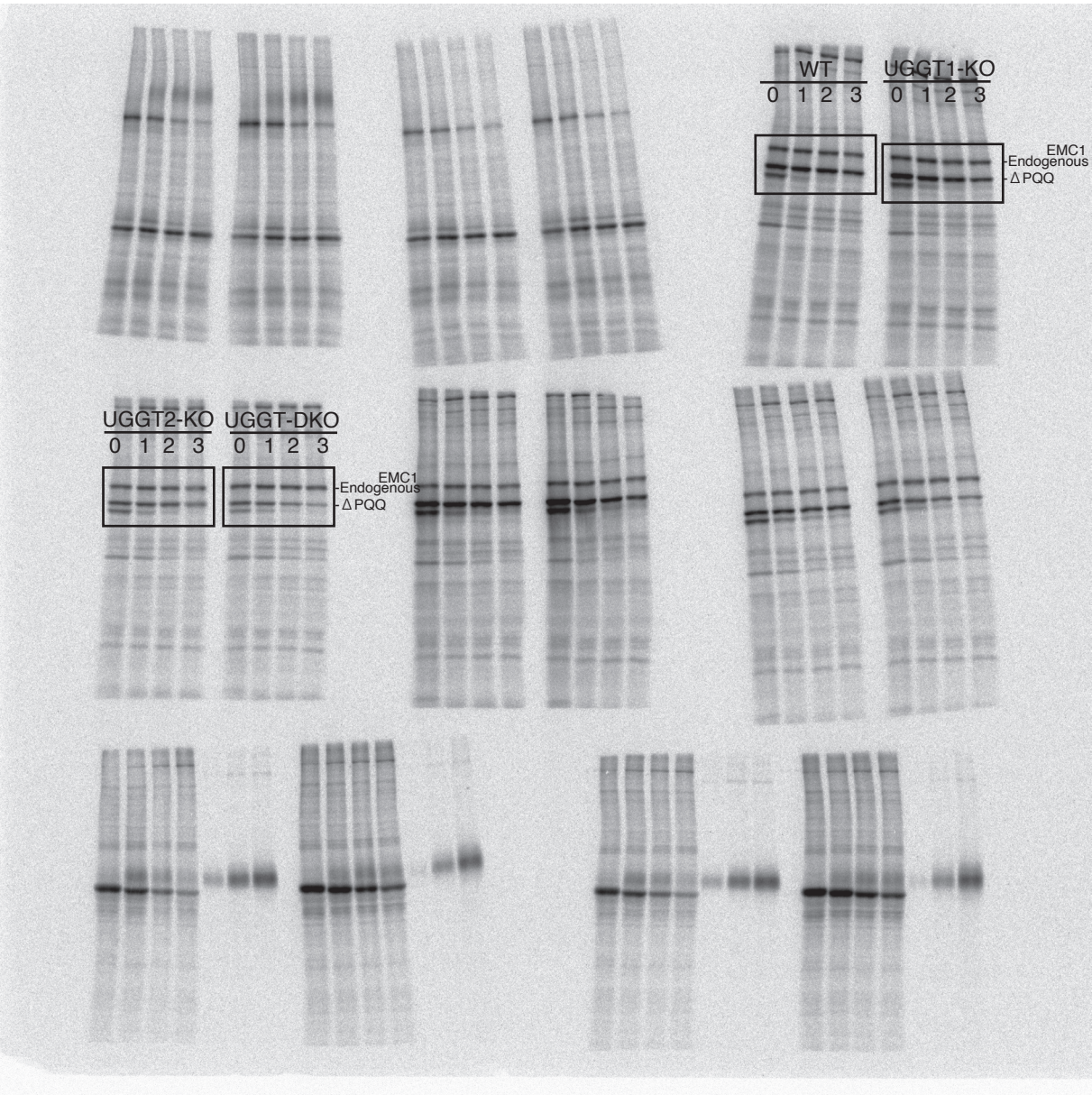

Supplement: Figure 3—source data 2. [file elife-93117-fig3-data2.zip › Fig. 3-Source data 2/Fig. 3F-Source data 2.pdf]

Fig. 3D Source data 2 Original membrane and gel corresponding to Fig3D.

Fig. 3D

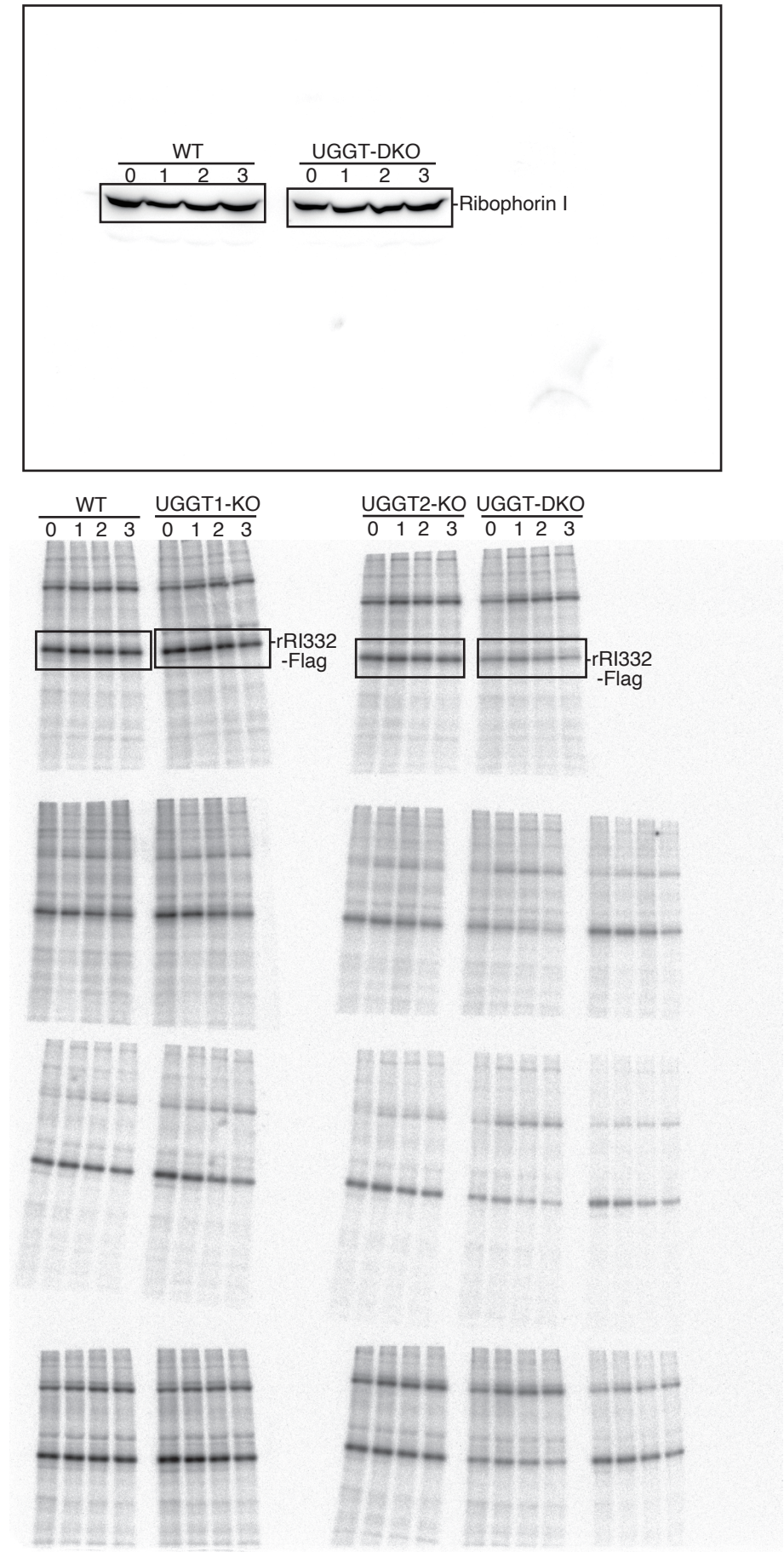

Supplement: Figure 3—source data 2. [file elife-93117-fig3-data2.zip › Fig. 3-Source data 2/Fig. 3D-Source data 2.pdf]

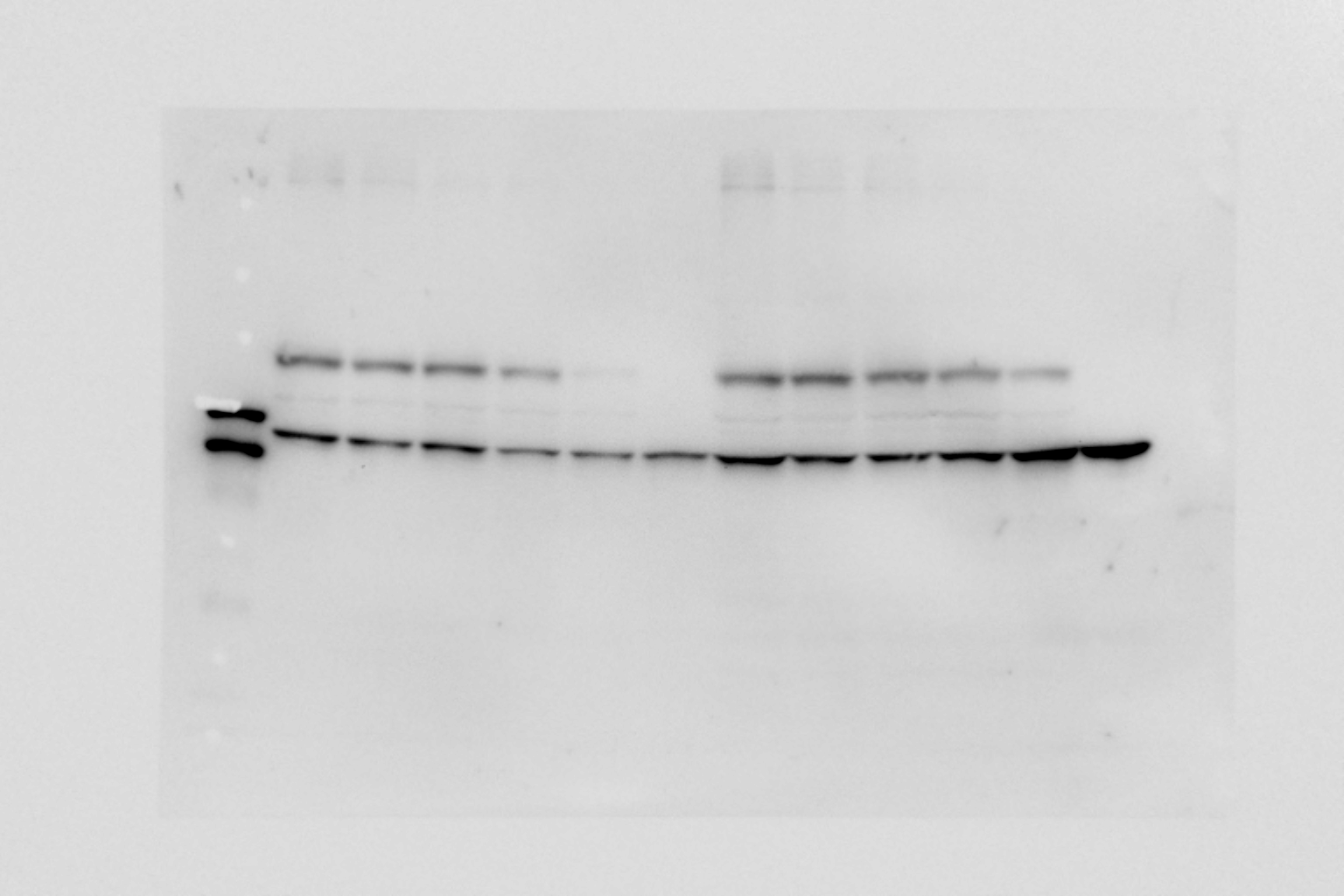

Supplement: Figure 4—source data 1. [file elife-93117-fig4-data1.zip › Fig. 4-Source data 1/Fig.4B-1-Source data 1.tif]

Fig. 4AB Source data 2 Original membranes corresponding to Fig4AB.

Fig. 4A

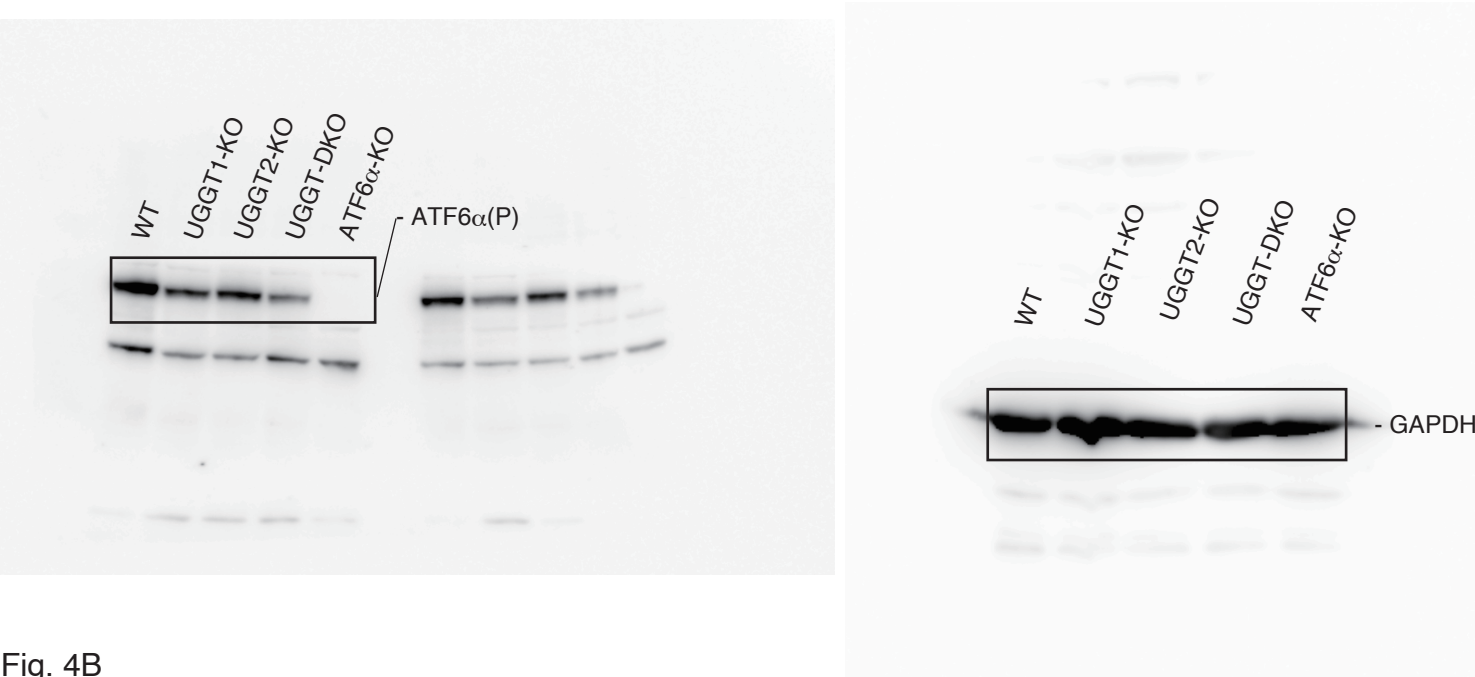

Fig. 4B

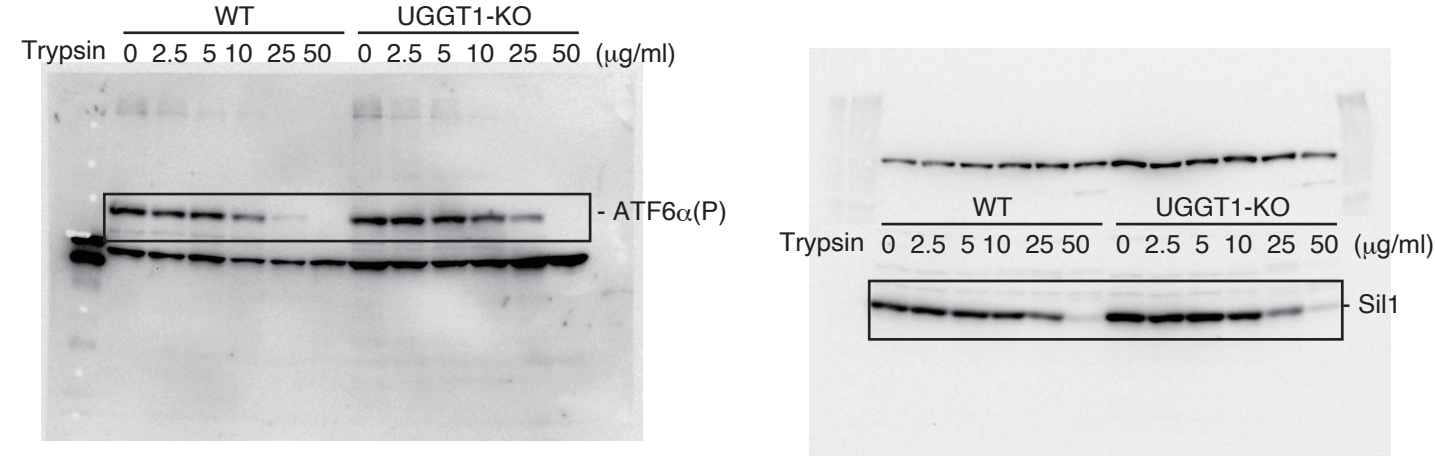

Supplement: Figure 4—source data 2. [file elife-93117-fig4-data2.zip › Fig. 4-Source data 2/Fig. 4AB-Source data 2.pdf]

Fig. 4C-E Source data 2 Original membranes corresponding to Fig4C-E.

Fig. 4C

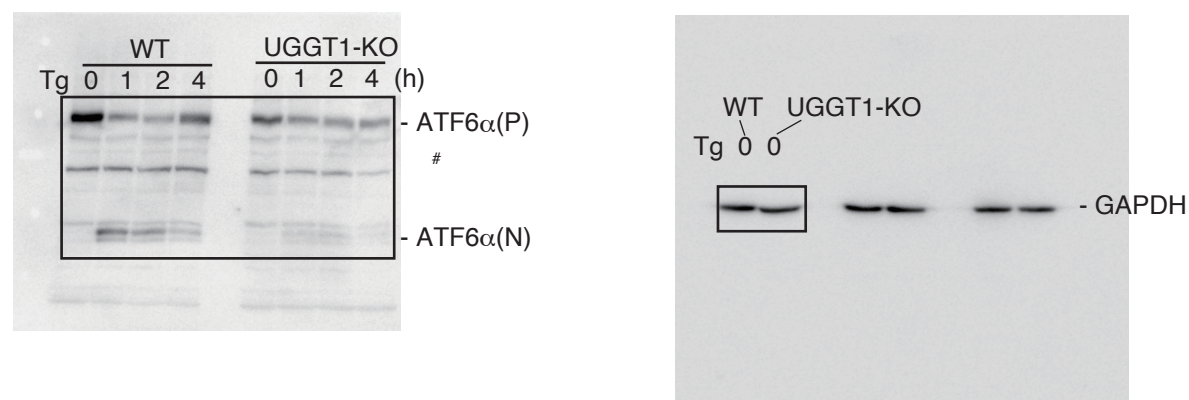

Fig. 4D

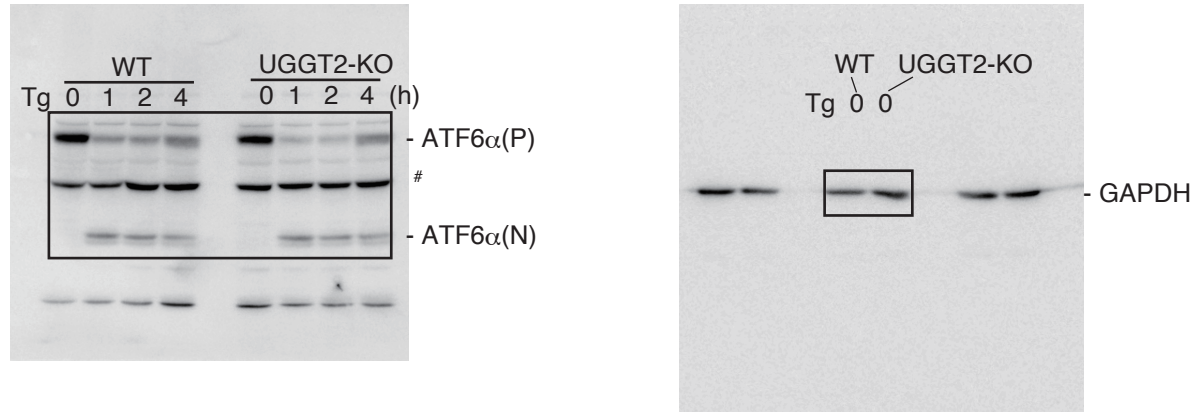

Fig. 4E

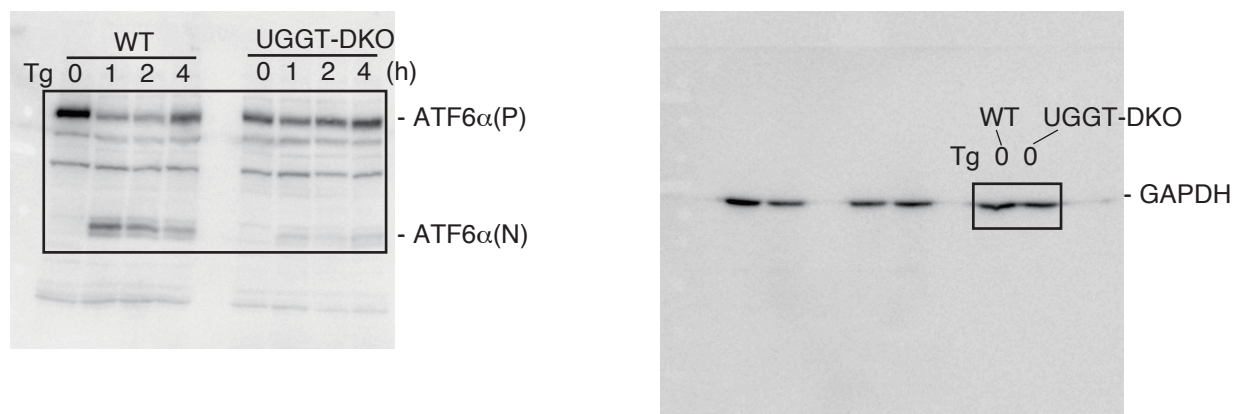

Supplement: Figure 4—source data 2. [file elife-93117-fig4-data2.zip › Fig. 4-Source data 2/Fig. 4C-E-Source data 2.pdf]
