# Supplementary material for: UGGT1-mediated reglucosylation of N-glycan competes with ER-associated degradation of unstable and misfolded glycoproteins
Source: eLife. 2024 Dec 10;12:RP93117. doi: 10.7554/eLife.93117 (PMC11630818; doi:10.7554/eLife.93117)

Fig. 1-Figure Supplement 1-Source data 2. Original gels corresponding to Fig1-Fig. Sup.1E-H.

Fig. Sup.1E

UGGT2-KO

UGGT1-KO UGGT-DKO

WT #1 #2 #1 #2 #1 #2

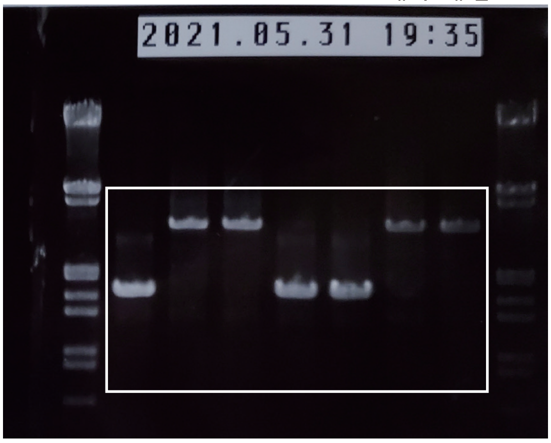

Fig. Sup.1F-1

UGGT2-KO

UGGT1-KO UGGT-DKO

WT #1 #2 #1 #2 #1 #2

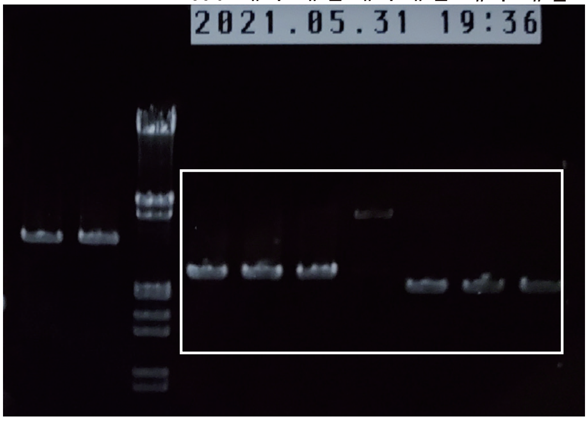

Fig. Sup.1F-2

UGGT2-KO

WT #2 #2 #1

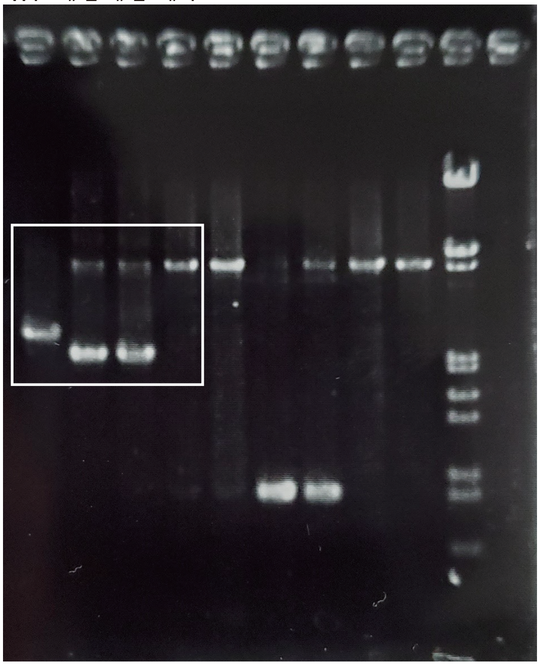

Fig. Sup.1G

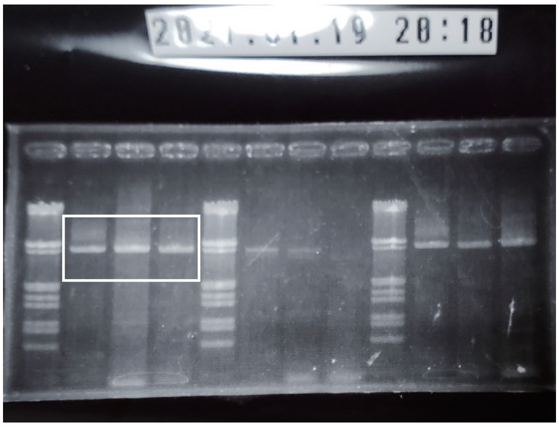

Fig. Sup.1H

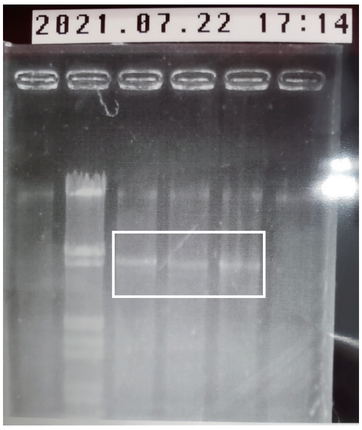

Supplement: Figure 1—figure supplement 1—source data 2. [file elife-93117-fig1-figsupp1-data2.pdf]
