# Supplementary material for: UGGT1-mediated reglucosylation of N-glycan competes with ER-associated degradation of unstable and misfolded glycoproteins
Source: eLife. 2024 Dec 10;12:RP93117. doi: 10.7554/eLife.93117 (PMC11630818; doi:10.7554/eLife.93117)

Fig. 1-Figure Supplement 2D-F Source data 2 Original membranes corresponding to Fig.1-Fig. Sup.2D-F.

Fig. Sup.2D

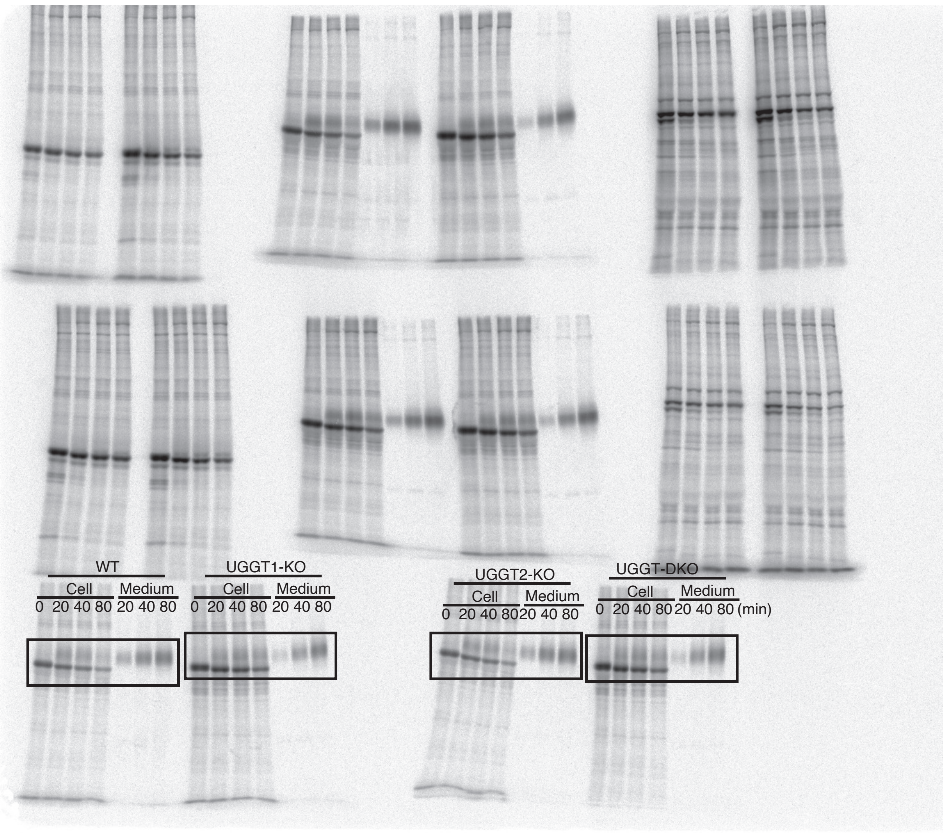

Fig. Sup.2E

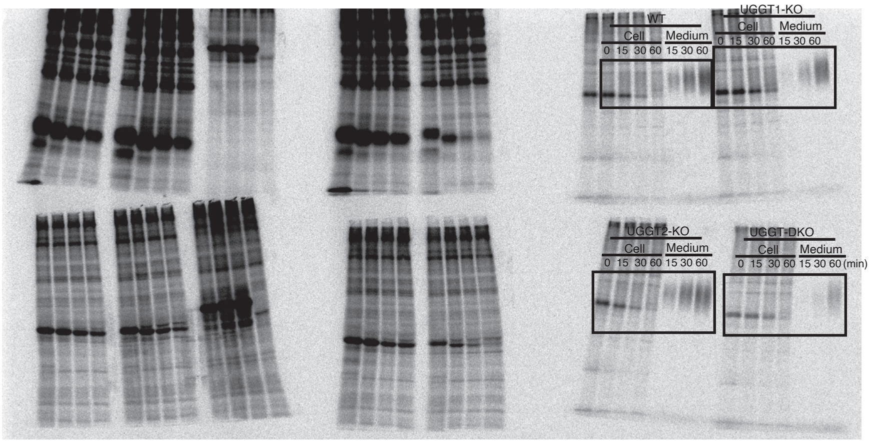

Fig. Sup.2F

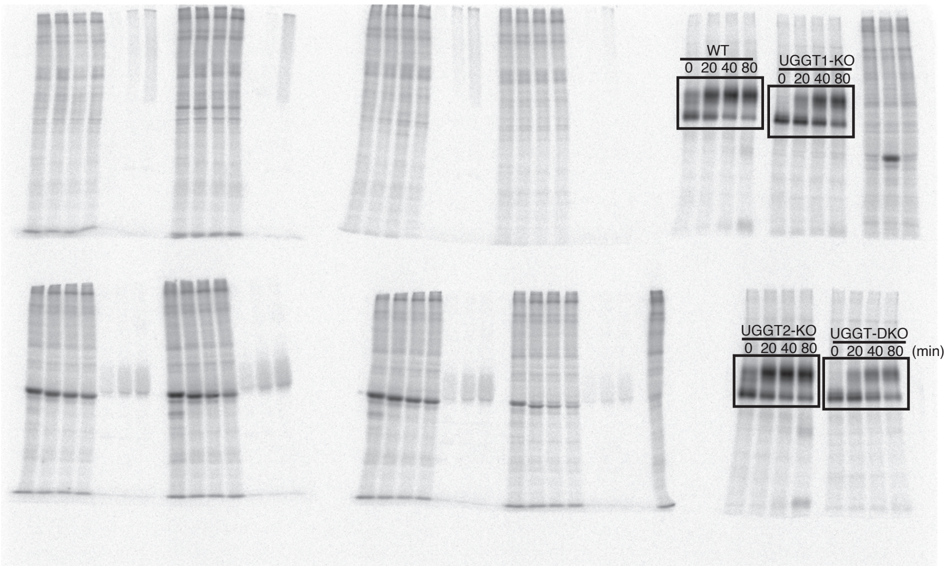

Supplement: Figure 1—figure supplement 2—source data 2. [file elife-93117-fig1-figsupp2-data2.zip › Fig1Supp2 D-F.pdf]
