# Supplementary material for: UGGT1-mediated reglucosylation of N-glycan competes with ER-associated degradation of unstable and misfolded glycoproteins
Source: eLife. 2024 Dec 10;12:RP93117. doi: 10.7554/eLife.93117 (PMC11630818; doi:10.7554/eLife.93117)

Fig. 1-Figure Supplement 2A-C Source data 2 Original membranes corresponding to Fig1-Fig. Sup.2A-2C.

Fig. Sup.2A

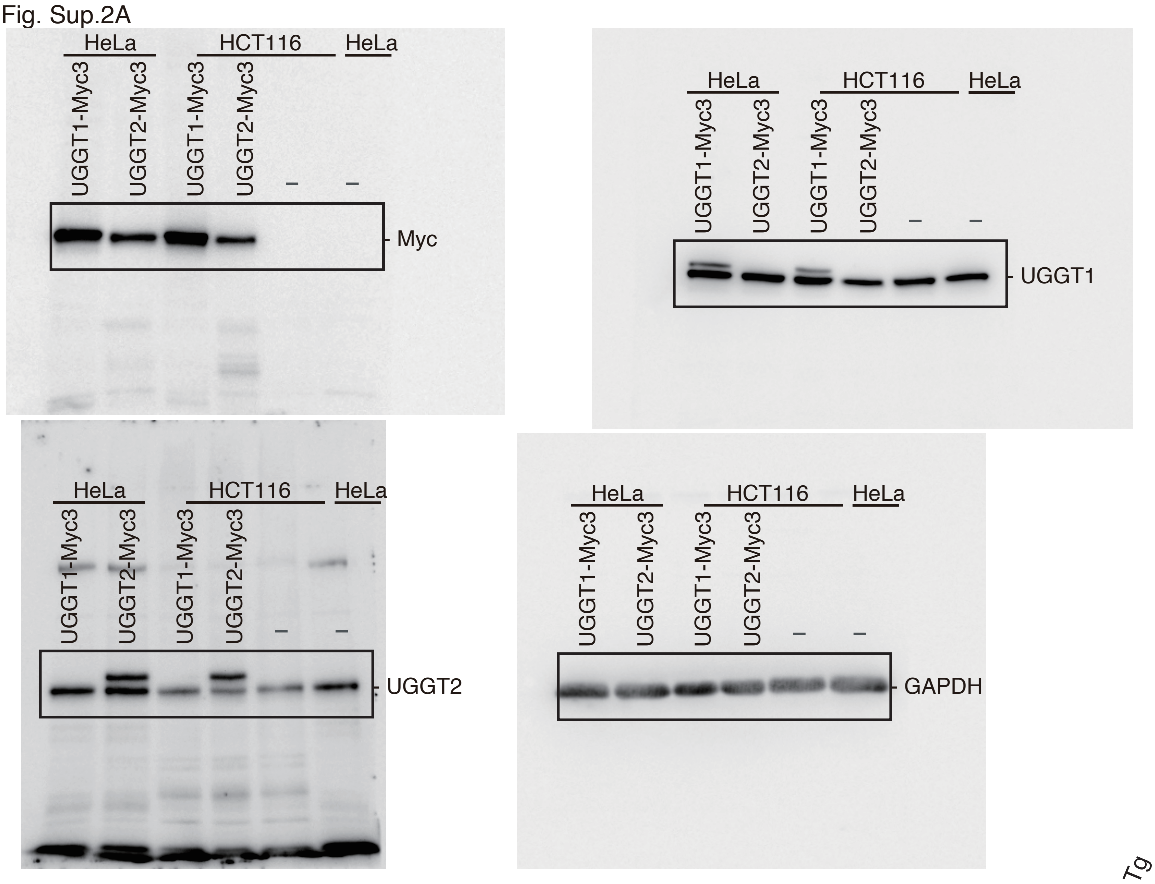

Fig. Sup.2B

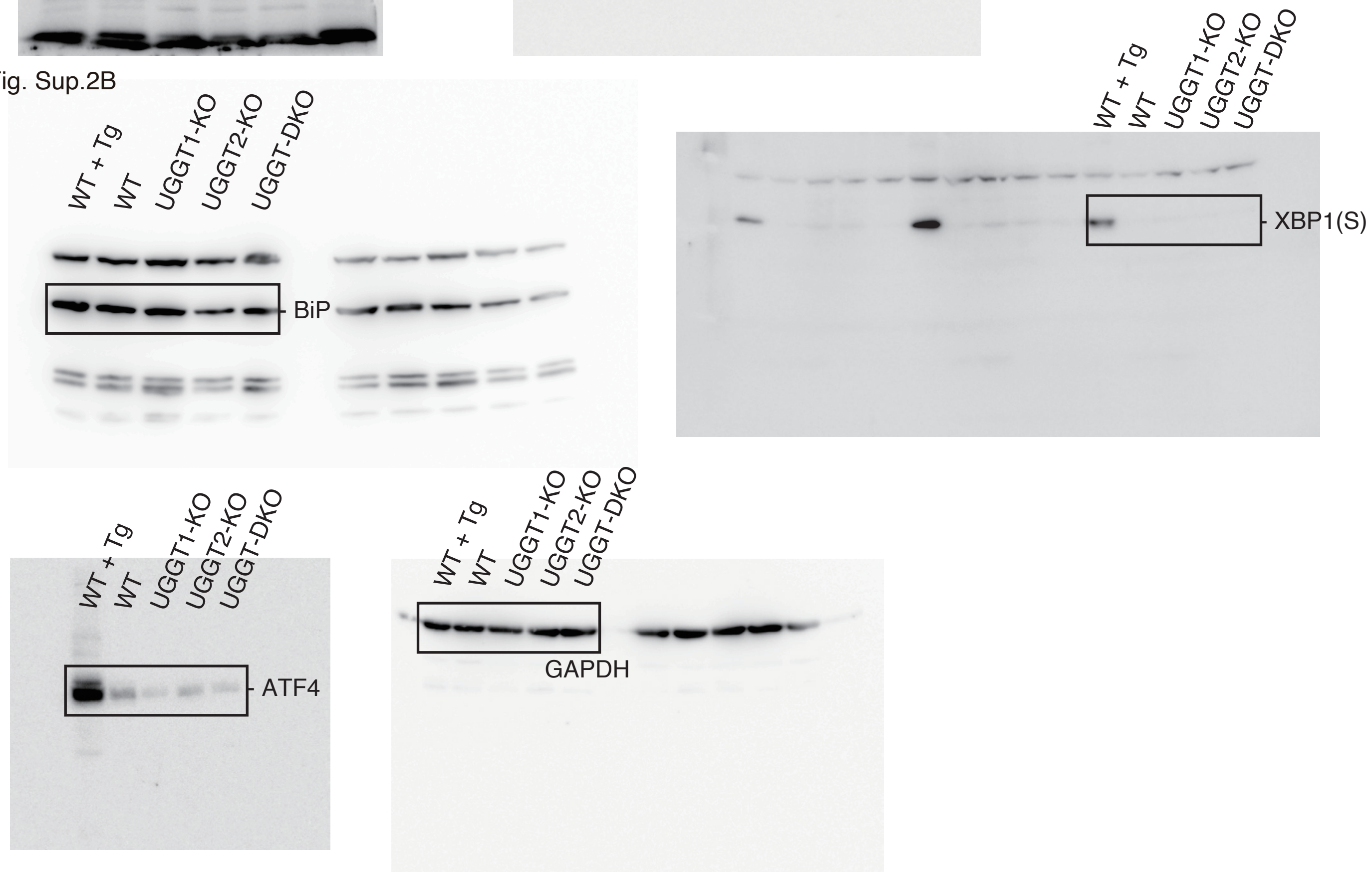

Fig. Sup.2C

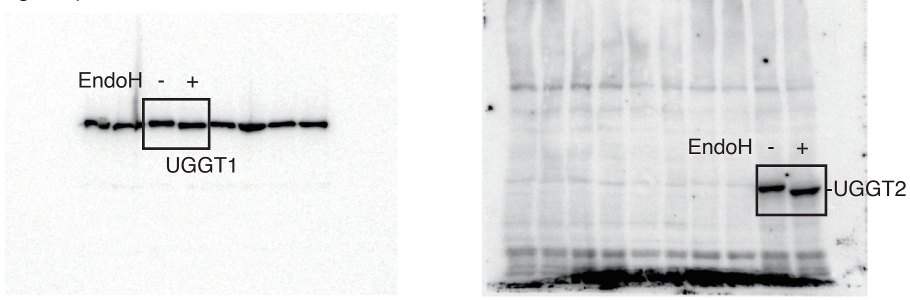

Supplement: Figure 1—figure supplement 2—source data 2. [file elife-93117-fig1-figsupp2-data2.zip › Fig1Supp2 A-C.pdf]
