# Supplementary material for: UGGT1-mediated reglucosylation of N-glycan competes with ER-associated degradation of unstable and misfolded glycoproteins
Source: eLife. 2024 Dec 10;12:RP93117. doi: 10.7554/eLife.93117 (PMC11630818; doi:10.7554/eLife.93117)
Supplement: Figure 1—figure supplement 3—source data 1. [file elife-93117-fig1-figsupp3-data1.zip › Fig. 1-Figure Supplement-3-Source data 1/Fig.1-Figure Supplement-3CD-Source data 1.pdf]

Campus notebooks contain the best ruled foolscap suitable for writing.

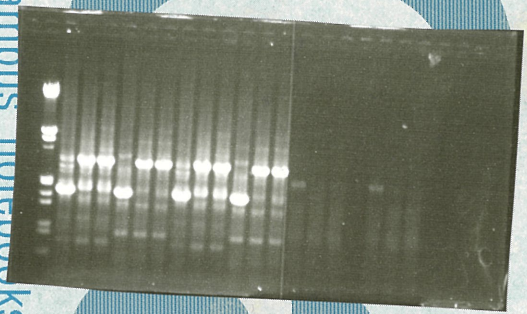

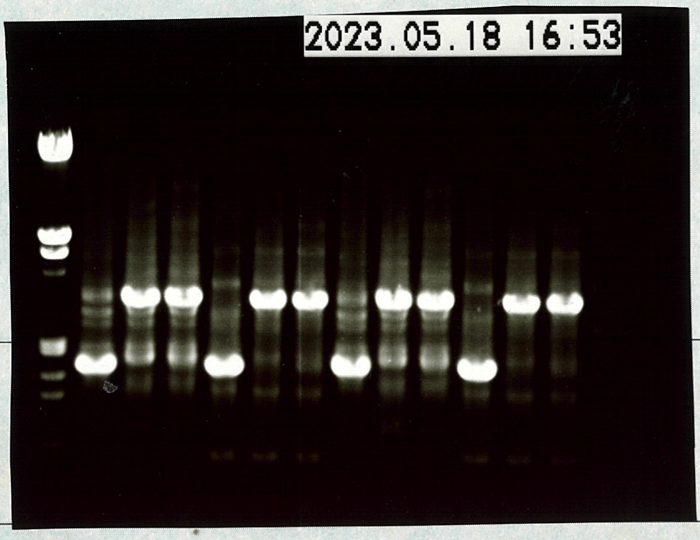

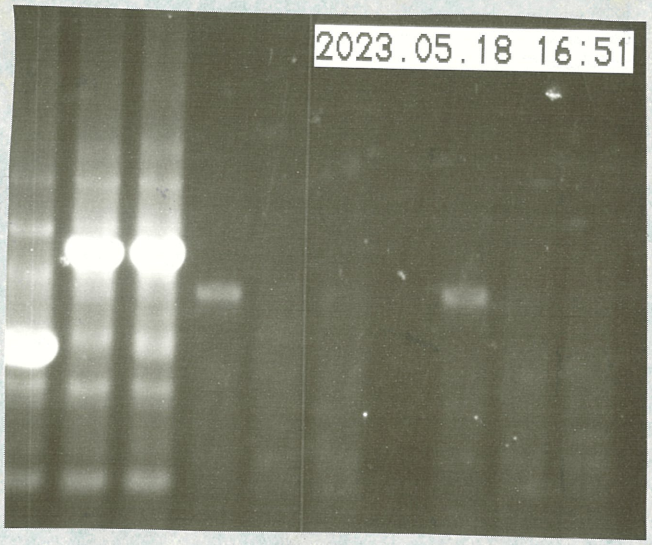

Campus®

NOTEBOOK

B 中横罫 6mm×41行 | 40枚 ノ−201B

KOKUYO
