# Supplementary material for: UGGT1-mediated reglucosylation of N-glycan competes with ER-associated degradation of unstable and misfolded glycoproteins
Source: eLife. 2024 Dec 10;12:RP93117. doi: 10.7554/eLife.93117 (PMC11630818; doi:10.7554/eLife.93117)
Supplement: Figure 1—figure supplement 3—source data 2. [file elife-93117-fig1-figsupp3-data2.pdf]

Fig. 1-Figure Supplement 3 Source data 2 Original gel or membrane corresponding to Fig1-Fig. Sup.3DE.

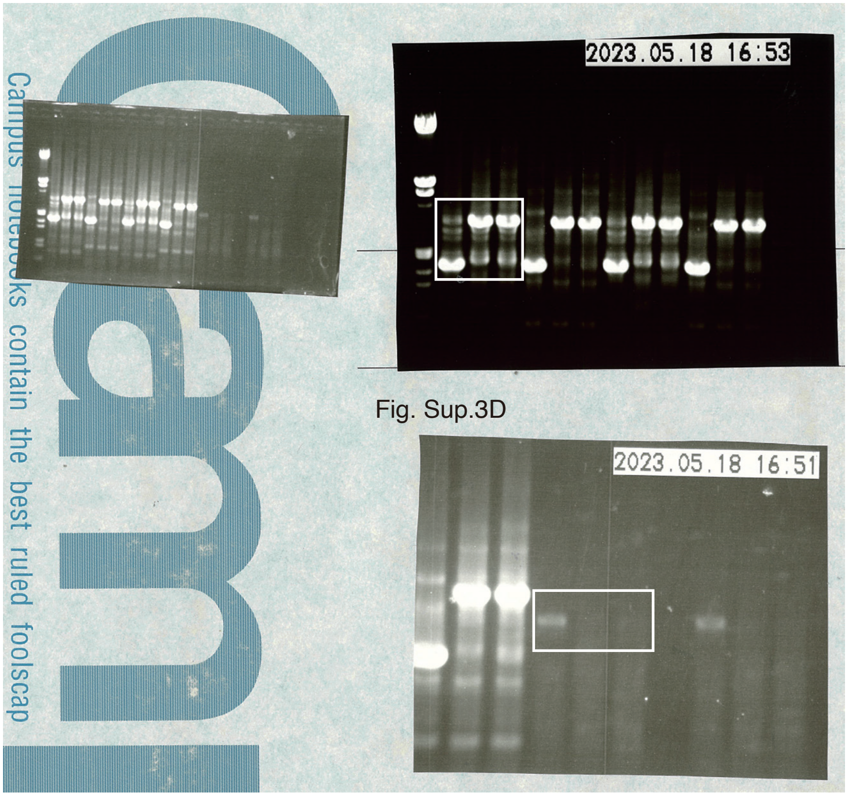

Fig. Sup.3D

Fig. Sup.3E

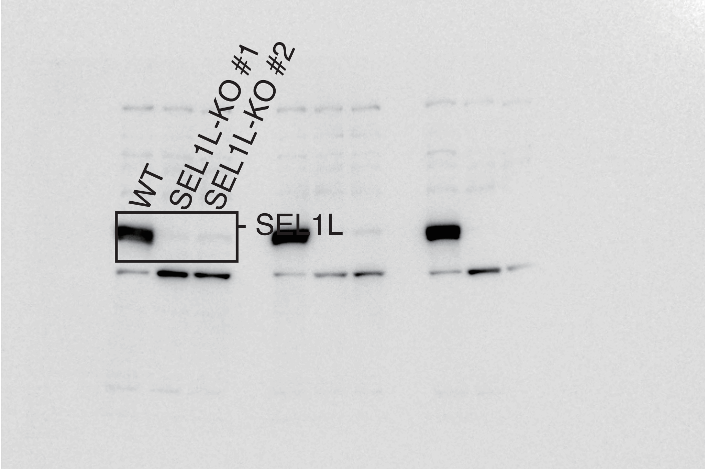

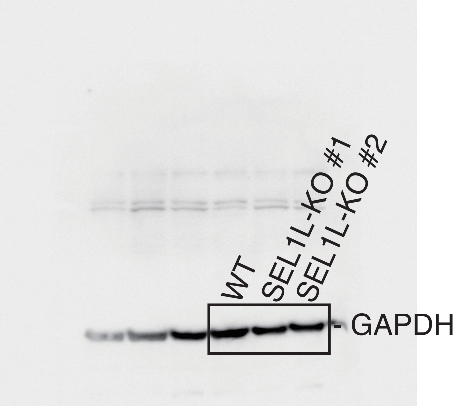

Fig. Sup.3F
