# Supplementary material for: UGGT1-mediated reglucosylation of N-glycan competes with ER-associated degradation of unstable and misfolded glycoproteins
Source: eLife. 2024 Dec 10;12:RP93117. doi: 10.7554/eLife.93117 (PMC11630818; doi:10.7554/eLife.93117)
Supplement: Figure 3—figure supplement 1—source data 2. [file elife-93117-fig3-figsupp1-data2.pdf]

Fig. 3-Figure Supplement 1 Source data 2 Original membranes corresponding to Fig3-Fig. Sup.3B.

Fig. Sup.3B

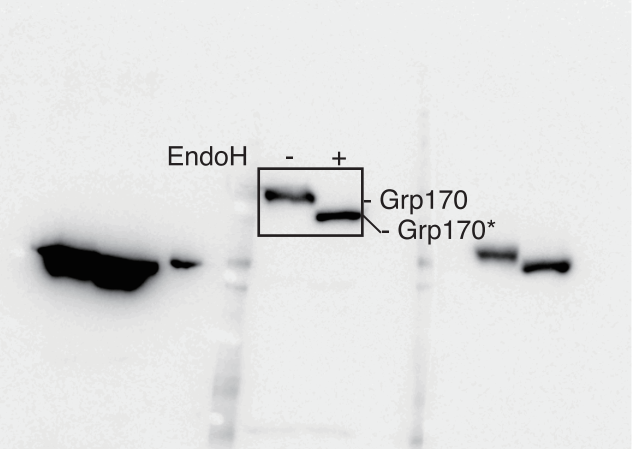

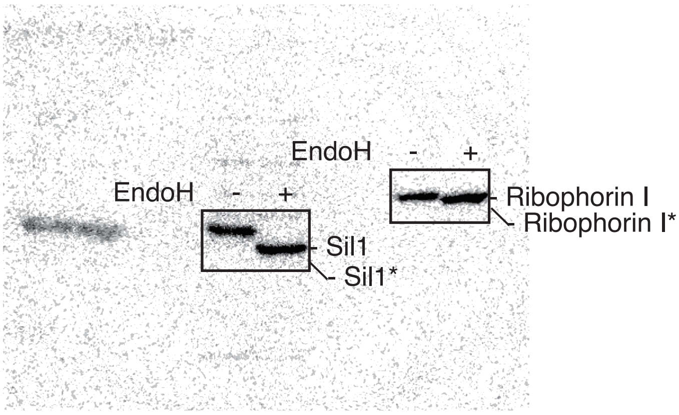

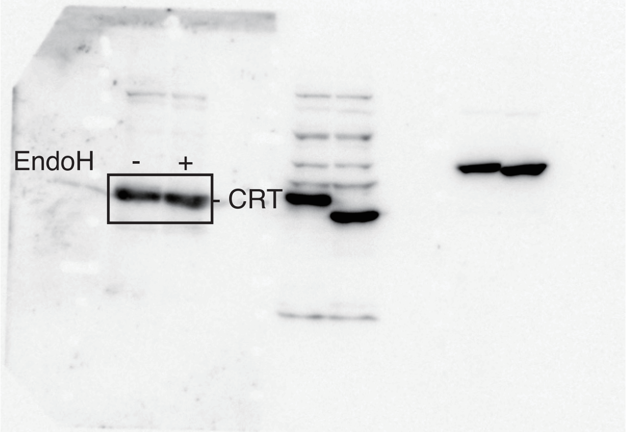

Fig. Sup.3F
